# Insights into the pH-dependent reactivity and substrate induced active-site reorganization of the non- heme iron enzyme *Cth*EgtB

**DOI:** 10.1007/s00775-026-02167-8

**Published:** 2026-07-30

**Authors:** Kassidy W. Rodriguez, Phuong Hong Ngoc Tao, Kiran Kurmi, Katlyn K. Meier

**Affiliations:** 1 Department of Chemistry, University of Miami, Coral Gables, FL 33146 USA; 2 Department of Molecular and Cellular Pharmacology, Sylvester Comprehensive Cancer Center, University of Miami Miller School of Medicine, Miami, FL 33136 USA; 3 The Frost Institute for Chemistry and Molecular Science, University of Miami, Miami, FL 33146 USA

**Keywords:** Non-heme Fe(II) enzyme, Sulfoxide synthase, O_2_ activation, Stopped flow kinetics, Affinity tag

## Abstract

Ergothioneine biosynthesis relies on the non-heme Fe(II) sulfoxide synthase EgtB, which couples O₂ activation to stereoselective C-S bond formation between trimethylhistidine (TMH; hercynine) and L-cysteine (L-Cys). Here, we utilize circular dichroism (CD), differential scanning fluorescence, light scattering, and stopped-flow spectroscopy to define how metal loading, substrate addition, oxygen availability, and pH shape the active site and catalysis of tetrameric EgtB from *Chloracidobacterium thermophilum* (*Cth*). Far-UV CD and thermal unfolding data confirm *Cth*EgtB’s stable, predominantly alpha helical secondary structure under all conditions studied, while Fe(II) and sequential substrate binding help to stabilize the enzyme. Visible CD demonstrates a distinct ligand-field reorganization upon sequential addition of Fe(II), L-Cys, and TMH to apo-*Cth*EgtB. Subsequent exposure to O₂ results in a shift of the ligand-field and charge-transfer features with associated appearance of a high energy feature consistent with the formation of sulfoxide product. The pH- and temperature-dependent kinetics of the reaction were monitored via stopped-flow absorption spectroscopy and show max activity at pH 7.0 and rates that scale with O₂ concentration, indicating tightly coupled O₂ binding and activation. Mass spectrometry confirms oxygen dependent formation of the hercynylcysteine sulfoxide product. Finally, we provide clear evidence that retention of a 6x N-terminal histidine-tag decreases the activity and stability of *Cth*EgtB, confirming that tagged constructs do not fully reproduce native behavior. Together, these results support a unified mechanism in which Fe(II), substrate binding, protonation environment around the active site, and O₂ availability poise the *Cth*EgtB active site for efficient oxygen activation and sulfoxide formation.

**Graphical Abstract:**

**Figure:**
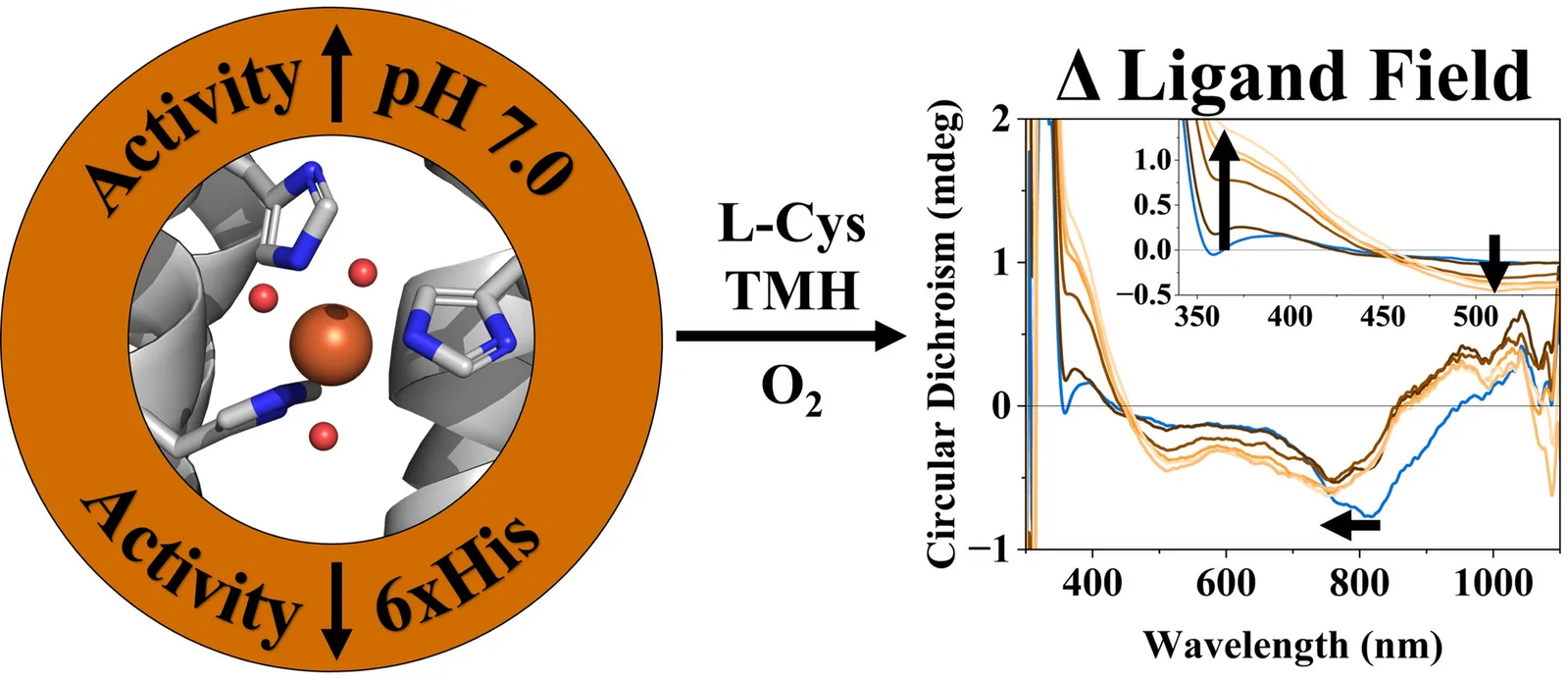

**Supplementary Information:**

The online version contains supplementary material available at https://doi.org/10.1007/s00775-026-02167-8.

## Introduction

**Scheme 1:**
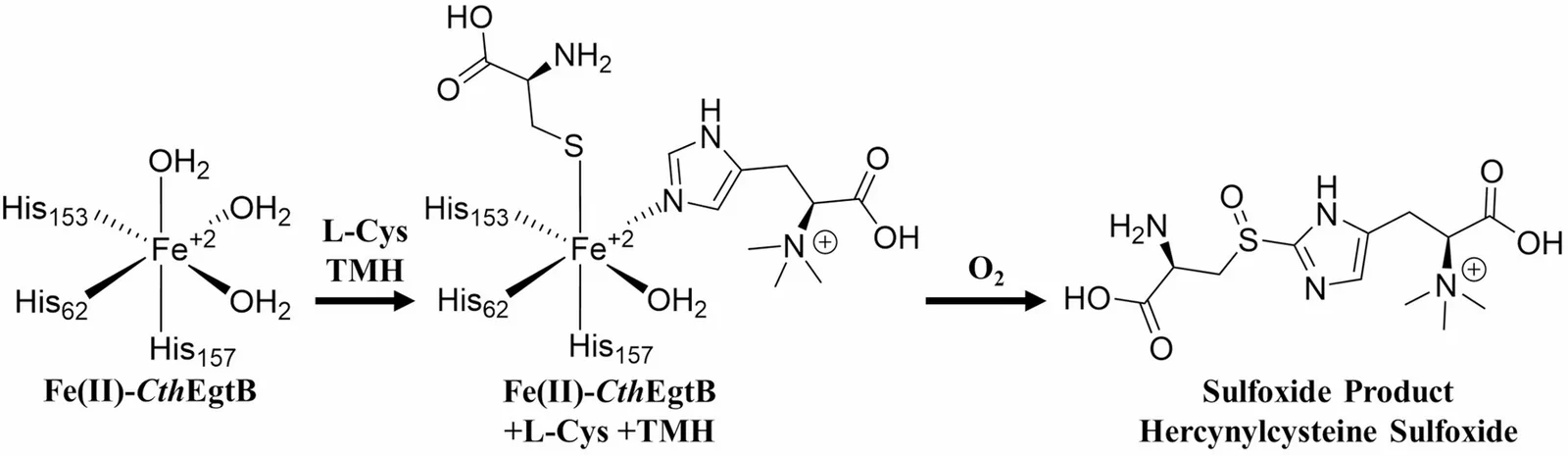
Non-heme Fe(II)-dependent C-S bond formation catalyzed by EgtB in EGT biosynthesis. *Cth*EgtB couples TMH and L-Cys in an O₂-dependent, stereoselective reaction to form hercynylcysteine sulfoxide, the sulfoxide product that channels the pathway toward EGT production. Amino acid numbering is based on crystal structure PDB ID: 6O6L

Ergothioneine (EGT) is a sulfur-containing antioxidant produced by fungi and select bacteria and is increasingly recognized for its roles in cellular redox protection and stress tolerance [1–6]. A defining step in EGT biosynthesis is catalyzed by EgtB, a non-heme Fe(II)-dependent sulfoxide synthase that couples O₂ activation to stereoselective C–S bond formation between trimethylhistidine (TMH; hercynine) and L-cysteine (L-Cys), generating hercynylcysteine sulfoxide as a committed intermediate in the pathway (Scheme 1) [7–12]. This transformation is chemically notable because it requires controlled activation of O₂ at a mononuclear Fe(II) center while simultaneously directing sulfur incorporation and sulfoxide formation without unproductive oxidation [13–17]. 

EgtB homologs share a conserved 3-His facial triad but differ in substrate preference, active-site architecture, oligomeric state, and proposed mechanisms for O₂ activation and product formation [8–11, 17, 18]. Previous structural, biochemical, spectroscopic, and computational studies have provided models for substrate orientation, Fe–O₂ reactivity, and C–S bond formation in EgtB and related sulfoxide synthases [8, 10, 13, 14, 19]. However, several mechanistic questions remain unresolved, specifically how Fe(II) loading and substrate binding reorganize the active-site ligand field, how O₂ availability affects the earliest catalytic steps, and how solution conditions such as pH, temperature, and retention of the 6xHis affinity tag tune enzyme–substrate reactivity [13, 15, 17, 20, 21]. *Chloracidobacterium thermophilum* EgtB (*Cth*EgtB) is a useful system for addressing these questions [9, 10, 21]. *Cth* is a thermophilic, microaerophilic chlorophotoheterotroph originally isolated from alkaline siliceous hot spring microbial mats. Although its native mat environment can be alkaline, growth physiology studies show that *Cth* strain Bᵀ grows over a broad pH range of 5.5–9.5, with relative growth assessed by bacteriochlorophyll *c* accumulation and an optimum near pH 7.0 [22–25]. It is a structurally characterized, tetrameric type II EgtB homolog with established Fe(II)- and O₂-dependent sulfoxide synthase activity [9, 10, 21]. Its tetrameric structure and active-site organization make it well suited for probing how local Fe-centered electronic changes couple to broader structural stability and catalytic behavior [9, 10, 21, 26–28]. 

Despite these advantages, key aspects of EgtB catalysis remain challenging to resolve because putative Fe–O₂ intermediates are short-lived and difficult to detect and accumulate under standard assay conditions [16, 17, 26, 29]. Furthermore, interpretation of prior *Cth*EgtB studies is complicated by differences in experimental design [9, 10, 21]. Many kinetic and mechanistic analyses have been conducted under aerobic conditions, with external reductants, without precise control of dissolved O₂, and using affinity-tagged constructs [9, 10]. These factors are especially important for studies of non-heme Fe enzymes, as O₂ concentration, protonation state, metal occupancy, and construct identity can each influence observed activity [26, 30–32]. In addition, previous studies used different conditions for structural (pH 6.5, no 6xHis tag) and kinetic measurements (pH 8.0, 6xHis tag), limiting direct comparison of structure, stability, and reactivity across studies. These differences are particularly relevant because the reported growth optimum of the source organism is near pH 7.0, leaving open the question of which pH conditions best support *Cth*EgtB stability and catalytic activity.

Here, we test the hypothesis that efficient *Cth*EgtB catalysis requires formation of a properly assembled Fe(II)- and substrate-bound state whose reactivity is governed by O₂ availability, protonation environment, and construct identity. Using anaerobically prepared samples and well-defined reaction conditions, we address the following questions: (i) how Fe(II), L-Cys, and TMH alter the active-site electronic structure, (ii) how dissolved O₂ concentration influences early product-associated signals; (iii) how pH and temperature define the enzyme’s catalytic operating window; and (iv) whether retention of an N-terminal 6xHis tag affects *Cth*EgtB activity and stability. To this end, we combine protein-induced chirality analysis via visible CD with complementary techniques including visible absorption, far-UV CD, nanoDSF, light scattering, stopped-flow absorption, and mass spectrometry. This integrated approach provides an experimentally controlled framework for understanding how metal loading and substrate binding reorganize the active site and impact enzyme stability, and how O₂ availability and environmental conditions influence *Cth*EgtB reactivity.

## Materials and methods

Additional details related to the Materials and Methods can be found in the Supplementary Information file.

### *Cth*EgtB construct, expression, and purification

The design of the *Cth*EgtB construct and the expression and purification procedures have been reported previously [21], with slight modifications that are described in the SI.

### Preparation of anaerobic enzyme samples

Purified *Cth*EgtB or 6xHis*Cth*EgtB was placed into sequential overnight dialysis against EDTA-containing buffer and Chelex^®^-treated HEPES buffer, sterile filtered, and adjusted to the desired concentration prior to Fe(II) reconstitution. Enzyme solutions were degassed on a lab Schlenk line using UHP argon and were transferred into an anaerobic glovebox (< 0.1 ppm O₂) for Fe(II) loading and substrate addition.

### Circular dichroism (CD)

Far-UV and visible CD spectra of *Cth*EgtB were collected using an Applied Photophysics Chirascan V100 spectrometer, with thermal unfolding monitored in the far-UV region (200–250 nm) from 20 to 90 °C to determine melting temperatures (Tₘ). Visible CD spectra (300–1100 nm) were acquired at 20 °C and analyzed using quadratic peak fitting in MATLAB to determine ligand-field transition maxima.

### Prometheus panta (nanoDSF)

Enzyme samples (2–4 µM) were clarified by centrifugation under anaerobic conditions, loaded into Prometheus-compatible capillaries (NanoTemper), sealed in the glovebox, and analyzed using a Prometheus Panta (NanoTemper) instrument.

### Stopped-flow kinetic measurements

Rapid-mixing UV–visible stopped-flow experiments were performed under anaerobic conditions using an SX20 spectrometer (Applied Photophysics) equipped with both single-wavelength absorbance and photodiode array detection to monitor the O₂-dependent reaction. For each condition, at least three independent stopped-flow shots were collected and averaged prior to analysis. The earliest experimentally accessible data point under the acquisition settings used in this study was 2 ms after mixing. Therefore, kinetic events occurring prior to 2 ms were not directly resolved, and no conclusions are drawn about species that may form and decay before this time point. All experiments were conducted in 20 mM buffer, with pH 6.5 and 7.0 measurements performed in 20 mM MOPS, pH 7.5 and 8.0 measurements performed in 20 mM HEPES, and pH 9.0 and 10.0 performed in 20 mM glycine. Anaerobically prepared Fe(II)-*Cth*EgtB + L-Cys + TMH complexes were rapidly mixed with oxygenated buffer at defined O₂ concentrations, and kinetic traces were obtained from at least three averaged replicate shots and analyzed across multiple enzyme concentrations. Additional information on buffer selection can be found in the SI.

### Oxygen-saturated buffer preparation and O₂ quantification

Dissolved O₂ concentrations in oxygenated buffers were quantified using previously established methods [33]. A methyl viologen-based colorimetric assay was utilized in which reduced methyl viologen (MV⁺•) is oxidized stoichiometrically by O₂, resulting in a loss of absorbance at 605 nm. All solutions were prepared anaerobically, and O₂ concentrations were calculated from absorbance changes using established extinction coefficients and protocols [33]. 

### LC-MS Sample preparation

All anaerobic sample preparation was performed in a glovebox maintained at ≤ 0.1 ppm O₂. Enzyme samples were prepared by adding Fe(II) to anaerobic *Cth*EgtB samples in a 1:1 molar ratio with enzyme. The samples contained 160 µM enzyme in 10 mM HEPES, pH 8.0. Following Fe(II) addition, L-cysteine and TMH were added at 10-fold molar excess relative to enzyme, giving final pre-reaction concentrations of 1600 µM L-cysteine and 1600 µM TMH.

Three LC-MS controls were prepared in parallel with the enzyme samples used for oxygen-dependent reactions: buffer only, containing 10 mM HEPES, pH 8.0; substrate/buffer control, containing 160 µM Fe(II), 1600 µM L-cysteine, 1600 µM TMH, and 10 mM HEPES, pH 8.0; and enzyme only, containing *Cth*EgtB and Fe(II) without added substrates. Enzyme reactions were initiated by mixing 500 µL of the anaerobic enzyme sample with 500 µL of oxygen-saturated buffer outside the glove box. Oxygen-saturated buffer was prepared by bubbling ultra-high-purity O₂ through the working buffer, 10 mM HEPES, pH 8.0. Reactions were allowed to proceed for 1000 s and were then quenched by addition of 100% ice-cold LC-MS-grade methanol to a final concentration of 80%.

### LC-MS analysis

Assay samples were prepared as described above. After adding methanol, samples were transferred to Eppendorf tubes, vortexed for 5 min at 4 °C, and centrifuged at maximum speed for 10 min at 4 °C. The supernatant was transferred directly to LC vials for LC-MS analysis. Samples were analyzed on a Thermo Scientific Orbitrap Exploris 240 mass spectrometer coupled to a Vanquish Flex UHPLC system. A column union was used to automate direct sample infusion from the LC autosampler. Mobile phase A consisted of LC-MS-grade water containing 20 mM ammonium hydrogen carbonate and 0.1% ammonium hydroxide, and mobile phase B consisted of 100% LC-MS-grade acetonitrile. An isocratic method was used at 50% B with a flow rate of 0.1 mL/min for 3 min. The mass spectrometer was operated in full-scan mode with heated electrospray ionization. Spray voltage was set to 3.6 kV in positive ion mode and 2.8 kV in negative ion mode. Sheath gas, auxiliary gas, and sweep gas were set to 40, 8, and 0.5 respectively. The ion transfer tube temperature was 275 °C, and the vaporizer temperature was 250 °C. MS1 spectra were acquired at a resolution of 90,000 with polarity switching from 0 to 3 min over an m/z range of 40–400. The RF lens was set to 70%, and automatic gain control was set to Standard. Raw data were processed using Thermo FreeStyle 1.8. Both substrate hercynine (C₉H₁₅N₃O₂) and product hercynylcysteine sulfoxide (C₁₂H₂₀N₄O₅S) were detected in positive ion mode. For product quantification, the extracted ion chromatogram (EIC) for m/z 333.1227, corresponding to the protonated molecular ion [M + H]⁺, was integrated. Hercynine was quantified using the extracted ion chromatogram for m/z 198.123.

## Results and discussion

### Spectroscopic evidence for stepwise enzyme stabilization via sequential ligand binding

Far-UV CD spectra of *Cth*EgtB (2–3 µM enzyme) collected at 20 °C (200–250 nm) showed pronounced minima at 211 and 227 nm, consistent with a predominantly α-helical structure (**Figure ****S1**). The far-UV CD spectra of the native enzyme before and after anaerobic preparation, following sequential addition of Fe(II), L-Cys, and TMH, and after exposure to O₂ in the presence of the sulfoxide product remained unchanged. Results obtained in two separate buffer systems, phosphate (20 mM dibasic phosphate, 20 mM NaCl) and HEPES (20 mM HEPES) at pH 8.0, indicate that *Cth*EgtB catalysis proceeds without global changes in secondary structure under all conditions used for subsequent measurements.

**Fig. 1:**
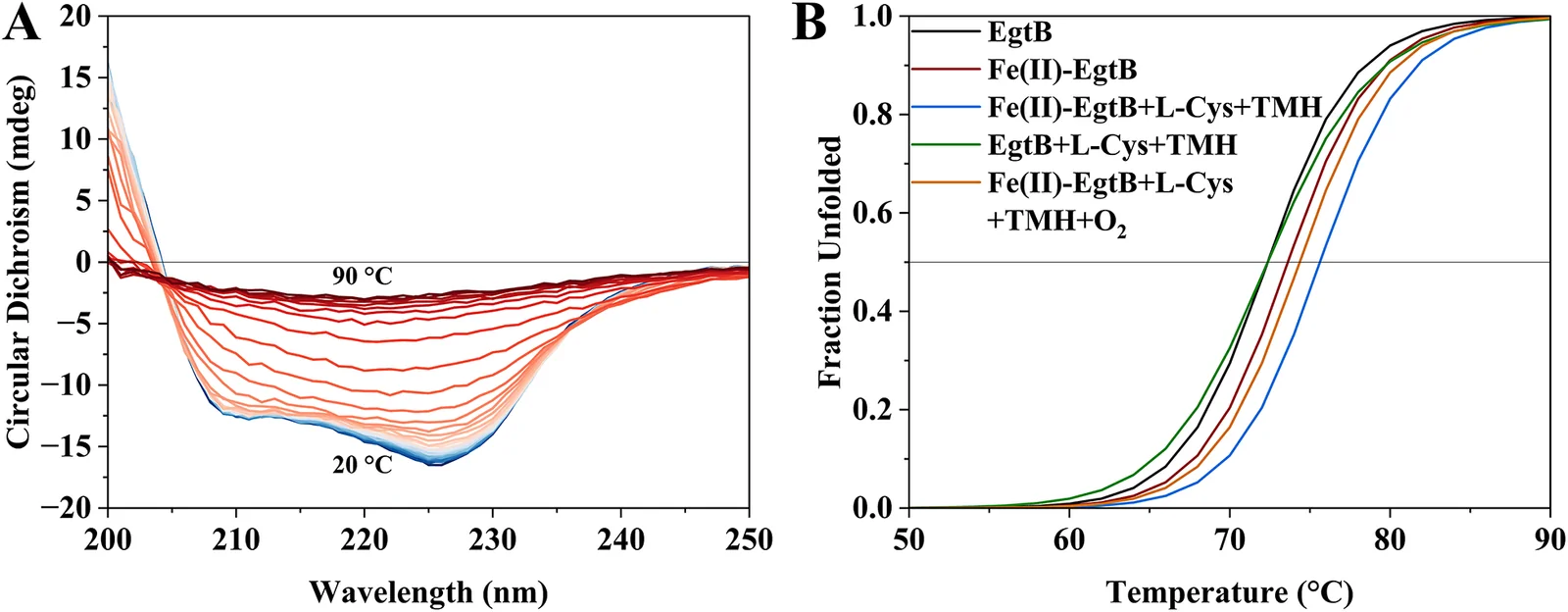
**(A)** Temperature-dependent far-UV CD spectra of Fe(II)-*Cth*EgtB collected under anaerobic conditions from 20 to 90 °C (blue to red) and from 200–250 nm. These spectra are used to monitor changes in secondary structure during thermal unfolding. **(B)** Sigmoidal thermal unfolding curves derived from the temperature-dependent CD signals and analyzed using Global3 software (Applied Photophysics). Shown are apo-*Cth*EgtB (black), Fe(II)-*Cth*EgtB (red), Fe(II)-*Cth*EgtB + L-Cys + TMH (blue), Fe(II)-*Cth*EgtB + L-Cys + TMH+O_2_ (orange), and *Cth*EgtB + L-Cys + TMH (green). The unfolding profiles were globally fit to a two-state unfolding model to determine the apparent melting temperature (T_m_)

Far-UV CD thermal unfolding experiments provide additional insight into how metal binding, substrate association, and product formation influence the secondary-structure stability of *Cth*EgtB (Fig. 1A-B, **Table ****S1**). The small but measurable difference in melting temperature (T_m_) determined for apo-*Cth*EgtB (72.36 °C) versus Fe(II)-*Cth*Egtb (73.62 °C), indicates that metal binding has a slight stabilizing effect on the folded enzyme. Addition of substrates L-Cys and TMH resulted in an additional increase in T_m_ to 75.62 °C. This progressive increase in thermal stability (total ΔT_m_ = + 3.26 °C) supports an ordered binding mechanism in which Fe(II) binding precedes substrate binding, with each ligand-binding step further organizing and stabilizing the overall secondary structure. Upon reaction of Fe(II)-*Cth*EgtB + L-Cys + TMH with O₂, conditions that generate the sulfoxide product, the T_m_ decreased by 1.26 °C, yet remained higher than that of the apo enzyme. Additionally, in the presence of only substrate without Fe(II) present, the T_m_ had no change from that of apo-*Cth*EgtB. Together, these CD data demonstrate that *Cth*EgtB secondary structure stability is modulated by metal and substrate interactions, and that ligand-driven active-site organization plays a critical role in stabilizing the enzyme. Notably, in every CD thermal unfolding experiment *Cth*EgtB does not unfold as a single cooperative unit but instead experiences domain-specific or motif-specific destabilization, where elements reported by the n→π* transition (227 nm, associated with alpha helical features stabilizing the tetramer) are perturbed earlier and less abruptly than those reflected in the π→π* (209 nm, alpha helical 2° features that are more tightly packed in each monomer) transition (**Figure ****S2**) [34–36]. 

To complement the CD-based thermal unfolding studies, *Cth*EgtB was further characterized by simultaneous measurement of intrinsic fluorescence (nanoDSF), static light scattering (SLS), and dynamic light scattering (DLS) during thermal denaturation (Prometheus Panta). Across all conditions tested, including aerobically and anaerobically prepared enzyme, Fe(II)-*Cth*EgtB in the absence and presence of substrates (L-Cys, TMH), and samples exposed to O₂, the nanoDSF-derived melting temperatures were statistically indistinguishable, yielding an average Tₘ = 71.89 ± 0.18 °C (**Table ****S2**). These data indicate that neither metal binding, substrate binding, nor anaerobic handling appreciably alters the global tertiary structural stability as monitored by intrinsic fluorescence.

The nanoDSF fluorescence traces revealed a gradual, linear increase in signal from 20 to 60 °C prior to the cooperative unfolding transition (**Figure ****S3**A-B). Although stabilization was not observed by nanoDSF, we did measure an increase in Tₘ upon Fe(II) and substrate binding in CD. While seemingly contradictory, our far-UV CD and nanoDSF data can be rationalized in terms of the structural features probed by the two methods. Thermal denaturation CD reports on the thermal sensitivity of secondary-structure features, in this case the alpha-helical features and the transition monitored at 227 nm, which can be influenced by tetramer organization. NanoDSF, by comparison, monitors local tertiary changes around aromatic residues. Taken together, complementary data from Panta and CD reveal that *Cth*EgtB unfolds through a multiphase process: early temperature-dependent rearrangement, followed by a cooperative global unfolding transition near ~ 72 °C, and finally aggregation through higher-order species detected by SLS (**Figure ****S3**C) and DLS (**Figure ****S3**D).

### Visible CD spectra provide insight into substrate-induced ligand field perturbations in *Cth*EgtB

**Fig. 2:**
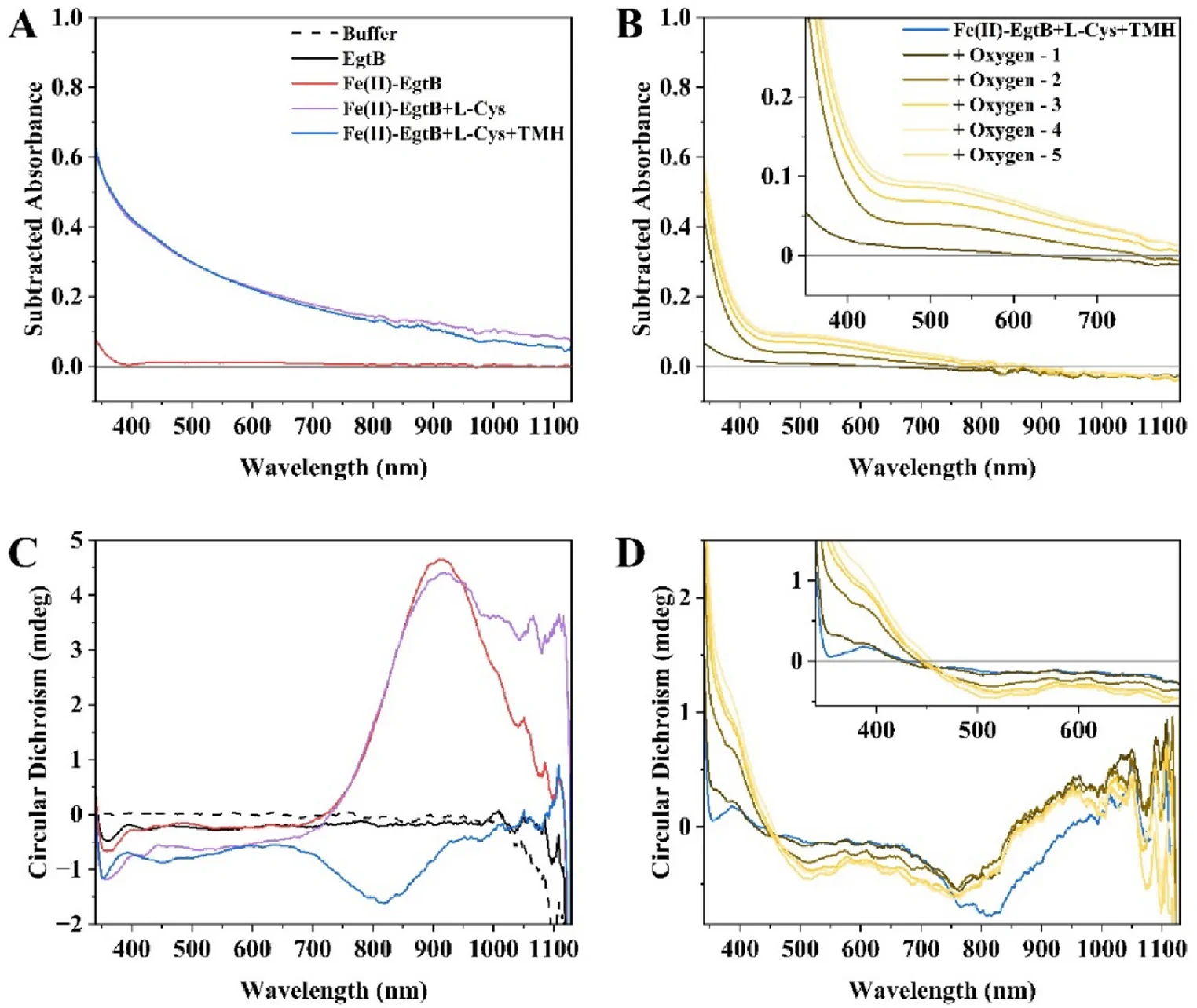
(**A**) Anaerobic visible absorption spectra of apo-*Cth*EgtB (black; 1.50 mM; 20 °C, 20 mM HEPES, pH 8.0), Fe(II)-*Cth*EgtB (red), and substrate-bound Fe(II)-*Cth*EgtB (purple = L-Cys only; blue = L-Cys + TMH). Spectra shown are difference spectra after subtracting the spectrum of apo-*Cth*EgtB. (**B**) Visible absorption spectra collected after five successive exposures (dark to light orange; labeled Oxygen-1 through Oxygen-5) of the preassembled Fe(II)-*Cth*EgtB + L-Cys + TMH complex (blue, 0.70 mM) to O₂. O₂ exposure results in the appearance of new absorbance features in the visible region. (**C**) Anaerobic visible CD spectra corresponding to the samples shown in panel (A) Apo-*Cth*EgtB (black) is CD-silent in the visible region, whereas addition of Fe(II), L-Cys, and TMH produce distinct ligand-field-dependent spectral changes. (**D**) Visible CD spectra collected after five successive O₂ exposures of the preassembled Fe(II)-*Cth*EgtB + L-Cys + TMH complex, corresponding to the absorption data shown in panel (B). O₂ exposure produces new visible CD features consistent with further reorganization of the Fe center during turnover. Absorption data are shown from 340 to 1130 nm because absorbance exceeded 2 AU below ~ 340 nm and detector noise increased near the long-wavelength cutoff (~ 1150 nm)

Visible CD and visible absorption spectroscopy data were simultaneously collected to monitor changes in the *Cth*EgtB active site upon Fe(II) and substrate addition under strictly anaerobic conditions (Fig. 2A, C). Spectra were acquired at 20 °C in 20 mM HEPES, pH 8.0, using anaerobically prepared buffer, apo-*Cth*EgtB, Fe(II)-*Cth*EgtB, Fe(II)-*Cth*EgtB + L-Cys, and Fe(II)-*Cth*EgtB + L-Cys + TMH with equimolar enzyme and substrate ratios (1:1:1:1, *Cth*EgtB: Fe(II): L-Cys: TMH). The visible absorption spectra shown in Fig. 2A are difference spectra generated by subtracting the apo-*Cth*EgtB spectrum, allowing Fe(II)- and substrate-dependent absorbance changes in the visible/near-IR region to be more clearly visualized. The corresponding raw and subtracted absorption spectra are provided in the Supplementary Information (**Figure ****S4**). Data are shown from 340 to 1130 nm because the raw absorbance exceeded 2 AU below ~ 340 nm. Additionally, the detector response and signal-to-noise decreased near the long-wavelength detector cutoff of the instrument at ~ 1150 nm.

The absorption data provide an important complement to the visible CD measurements and confirm that Fe(II) and substrate addition produce measurable changes in the electronic environment of the metal center. However, as expected for weak Fe(II)-centered ligand-field transitions, the absorbance features in the visible/near-IR region are broad and weak intensity. In contrast, visible CD provides more resolved spectral changes because the Fe center is embedded in the chiral protein environment. Thus, visible CD is particularly useful for monitoring ligand-field perturbations in *Cth*EgtB that are less apparent by conventional absorption spectroscopy alone.

As expected for a metal-free enzyme, apo-*Cth*EgtB exhibited no CD-active features in the visible energy range (Fig. 2C, **black**). Addition of Fe(II) produced a new positive intensity band at 913 nm (Fig. 2C, **red**), consistent with a d-d ligand-field transition from a high-spin Fe(II) center in a non-centrosymmetric protein environment [26, 37–39]. Addition of L-Cys to Fe(II)-*Cth*EgtB resulted in a slight red shift of this feature to 920 nm (Fig. 2C, **purple**), indicating that L-Cys perturbs the Fe(II) coordination environment. This change is consistent with weak or transient interaction of L-Cys with the metal center, or with substrate-induced reorganization of the active site near the Fe(II) center [28, 39]. 

Following addition of TMH, the positive feature near 920 nm disappeared and a new negative intensity feature was observed at 813 nm (Fig. 2C, **blue**). This shift to higher energy is consistent with an increase in ligand-field strength and a change in the geometric or electronic structure of the Fe(II) site. These changes are consistent with TMH binding through the imidazole group, displacement or reorganization of a solvent ligand, and formation of the complete enzyme–substrate complex, Fe(II)-*Cth*EgtB + L-Cys + TMH. Together, the anaerobic absorption and visible CD data show that sequential addition of Fe(II), L-Cys, and TMH produces measurable active-site reorganization without changes in global secondary structure.

### Probing the O₂-reactivity of Fe(II)-*Cth*EgtB + L-Cys + TMH by visible CD

To characterize changes in the Fe coordination environment upon reaction with O₂ from air, the anaerobically prepared Fe(II)-*Cth*EgtB + L-Cys + TMH complex was exposed to atmospheric O₂ in five controlled steps and monitored by both visible absorption and visible CD spectroscopy (Fig. 2B, D). The corresponding subtracted absorption spectra show the appearance of new O₂-dependent absorbance features in the visible region (Fig. 2B), supporting further electronic reorganization of the enzyme–substrate complex upon O₂ exposure. Raw and subtracted absorption spectra for these O₂-exposed samples are provided in the Supporting Information (**Figure ****S4**).

The visible CD spectra showed progressive and reproducible changes after each brief O₂ exposure (Fig. 2D). Upon the first O₂ exposure, the negative intensity feature associated with the anaerobic Fe(II)-*Cth*EgtB + L-Cys + TMH complex shifted to higher energy and resolved into two distinct negative intensity features at approximately 756 and 518 nm. This change was accompanied by the appearance of a new positive intensity band near 390 nm. Subsequent O₂ exposures resulted in a steady increase in the intensity of the 518 nm feature, indicating continued O₂-dependent reorganization of the Fe-centered ligand field.

Because the reaction was initiated by stepwise O₂ exposure and was not optimized to trap short-lived intermediates, we do not assign these spectra to a discrete Fe–O₂ intermediate. Instead, the visible CD and absorption changes are interpreted more conservatively as reflecting an O₂-reacted mixture of species generated from the preassembled enzyme–substrate complex. This mixture likely includes contributions from unreacted Fe(II)-*Cth*EgtB + L-Cys + TMH (ES) and product-associated states, either enzyme-bound or released, under conditions where O₂ exposure is limiting relative to the preassembled ES complex. The presence of both long-wavelength ligand-field/LMCT features and near-UV charge-transfer features is consistent with substantial reorganization of the Fe environment during O₂-dependent turnover.

Importantly, the O₂-dependent visible CD changes are more clearly resolved than the corresponding absorption features, highlighting the advantage of visible CD for monitoring Fe-centered electronic and geometric changes in *Cth*EgtB. Because the Fe site is embedded in a chiral protein environment, visible CD provides a sensitive readout of ligand-field perturbations that are difficult to observe by conventional absorption spectroscopy alone. To the best of our knowledge, this is the first report using visible CD to characterize the *Cth*EgtB active site, the anaerobic Fe(II)-*Cth*EgtB + L-Cys + TMH enzyme–substrate complex, and its reaction with O₂.

### Kinetic characterization of *Cth*EgtB catalysis by stopped flow absorption spectroscopy

It is known that *Cth*EgtB requires O₂ to form its sulfoxide product [9, 10]. To further define how O₂ availability, pH, temperature, and retention of a 6xHis tag influence *Cth*EgtB catalysis, we monitored sulfoxide formation using time-resolved stopped-flow absorbance spectroscopy. All solutions were prepared under strictly anaerobic conditions in a glovebox (O₂ < 0.1 ppm) except for the oxygenated buffers used to trigger the reaction. Dissolved O₂ concentrations were rigorously quantified (see Materials and Methods in the SI). Data shown are after subtraction of anaerobically prepared Fe(II)-*Cth*EgtB + L-Cys + TMH (ES; syringe 1) mixed with anaerobic buffer (syringe 2) (**Figure ****S5**A-B). Catalysis was initiated by mixing the pre-formed ES complex with buffer equilibrated to defined O₂ concentrations and was tracked via changes in absorption spectra (**Figure ****S5**A,** C**). In all experiments, Fe(II) was maintained at a 1:1 molar ratio with enzyme (1 monomer : 1 Fe(II)), while TMH and L-Cys were present in excess (1:10 molar ratio) to ensure pseudo-first-order conditions for kinetic analysis.

**Fig. 3:**
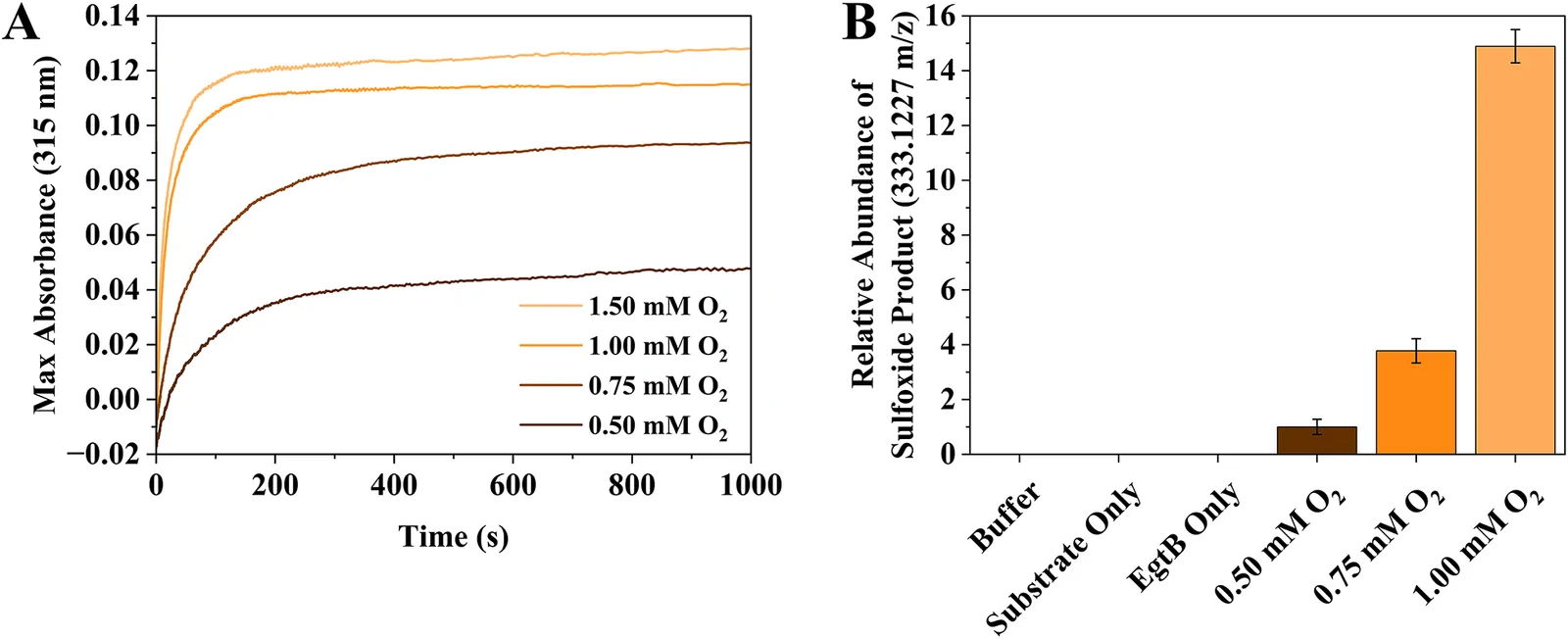
O₂-dependent *Cth*EgtB turnover monitored by stopped-flow absorbance and LC-MS product detection. (**A**) Time-resolved stopped-flow absorbance traces monitoring Fe(II)-*Cth*EgtB-catalyzed reaction at 315.5 nm as a function of dissolved O₂ concentration. Anaerobically prepared Fe(II)-*Cth*EgtB was preincubated with L-Cys and TMH to form the enzyme–substrate complex and then rapidly mixed with O₂-equilibrated buffer. Enzyme, Fe(II), and substrate concentrations were held constant while the dissolved O₂ concentration was varied from 0.50 to 1.50 mM. Increasing O₂ concentration produced progressively faster increases in absorbance and higher endpoint signal intensities. **(B)** LC-MS analysis of hercynylcysteine sulfoxide formation under matched O₂-initiated turnover conditions. Product abundance was quantified from the extracted ion chromatogram for the protonated sulfoxide product, [M + H]⁺, at *m/z* 333.1227. No sulfoxide product was detected in buffer-only, substrate-only, or enzyme-only controls, whereas O₂-initiated enzyme reactions showed increasing product abundance at 0.50, 0.75, and 1.00 mM O₂. Bars represent the mean relative abundance from technical triplicates, and error bars represent standard deviation

Upon exposure of anaerobic ES to O₂, we observed an immediate increase in absorbance at 315.5 nm. We interpret this feature as a reaction-associated absorbance change under turnover conditions. Samples were prepared under the same conditions for orthogonal LC-MS/HPLC analysis. These samples were quenched by methanol addition at t = 1000 s, corresponding to the longest stopped-flow timepoint. These experiments confirm depletion of TMH and formation of the hercynylcysteine sulfoxide species under O_2_-initiated turnover conditions. The sulfoxide product was not detected in the anerobic controls (Fig. 3B, S6). This reaction was so efficient that we could not detect any chromophoric intermediates from the earliest accessible timepoint of 2 ms. Product formation therefore begins within the instrument dead time (2 ms), consistent with a pre-organized active site in which O₂ binding and the first irreversible chemical step are tightly coupled.

To evaluate whether O_2_ concentration could be used to tease out relevant catalytic intermediates, we kept the enzyme and substrate concentrations constant (1 mM enzyme, 1:1 Fe(II): enzyme, and 10-fold excess TMH and L-Cys) and systematically varied the dissolved O₂ concentrations over the range from 0.5mM to 1.5mM (pre-mixing). Increasing the O₂ concentration produced progressively steeper initial slopes in the stopped-flow absorbance traces at 315.5 nm (Fig. 3A), indicating faster initial velocities. In addition to influencing reaction rates, the O₂ concentration also determined the overall extent of sulfoxide product formation, as increasing dissolved O₂ concentrations (0.5–1.5 mM) resulted in correspondingly higher absorbance maxima at 315.5 nm and larger relative abundance of hercynylcysteine sulfoxide as detected by LC-MS/HPLC (Fig. 3B). These observations show that the initial rate of the 315.5 nm signal is O₂-dependent, however, the endpoint absorbance is not interpreted as a direct stoichiometric measure of the amount of O₂ delivered.

Taken together, the rapid product formation, the O₂-dependent increases in initial rates, and the absence of detectable intermediates support a mechanistic model in which O₂ binding and activation occur rapidly upon encounter with the ES complex. Importantly, while mechanistic proposals for EgtB have invoked Fe/O intermediates, and although there is evidence that exposure of the anaerobic ES complex to O₂ alters the ligand-field environment, we did not detect any Fe/O intermediates by stopped-flow spectroscopy. We were, however, able to provide a rigorous analysis of the O_2_-dependent rate of product formation for *Cth*EgtB, which was previously absent from the literature.

### The catalytic rate and thermal responsiveness of *Cth*EgtB is optimal at pH 7.0

**Fig. 4:**
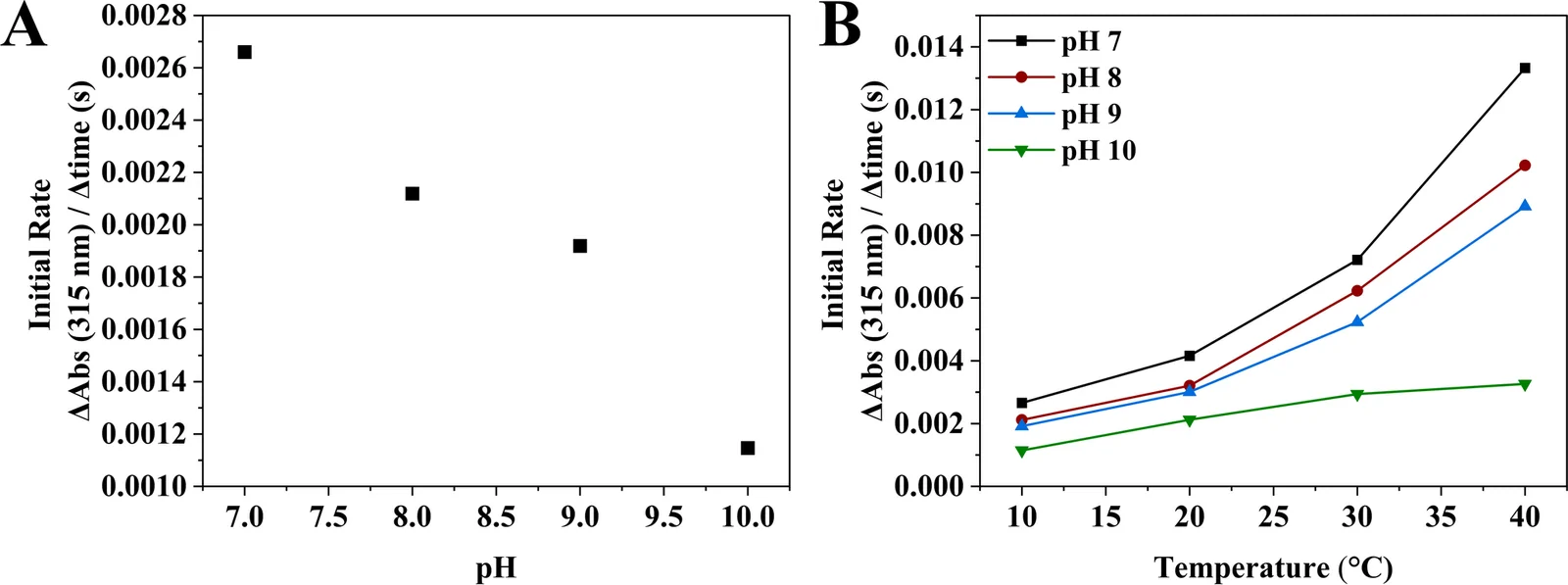
**(A)** pH dependence of *Cth*EgtB instantaneous velocity (v₀) measured at 10 °C. Initial rates of sulfoxide product formation were determined across pH 7.0–10.0 using three enzyme concentrations (~ 20–60 µM) with TMH and L-Cys present at a tenfold molar excess and dissolved O₂ at ~ 1.5 mM. Initial velocities were extracted from the linear region corresponding to the first ~ 10% of product formation. **(B)** Temperature dependence of *Cth*EgtB instantaneous velocity as a function of pH. Initial rates were measured over the temperature range from 10–40 °C in 10 °C increments. Product formation was monitored at the sulfoxide product λ_max_, and v₀ values were obtained from initial slopes following anaerobic mixing and defined O₂ addition

To define how solution pH and temperature influence the chemical steps governing *Cth*EgtB catalysis, instantaneous rates of sulfoxide formation were quantified across a pH range of 7.0–10.0 and temperatures spanning 10–40 °C. Reactions were performed under enzyme-limited conditions using low *Cth*EgtB concentrations (~ 20–60 µM) with all reactants present in excess, including TMH and L-Cys (10-fold molar excess) and dissolved O₂ (~ 1.5 mM). Each experiment was conducted for three enzyme concentrations to verify enzymatic behavior and rule out concentration dependent kinetics. The conditions were selected to report on intrinsic catalytic events at the Fe(II) active site and to allow analysis within the linear regime corresponding to the first ~ 10% of product formation. We maintained a high O₂:*Cth*EgtB ratio to minimize oxygen-limiting effects and isolate the impact of pH and temperature on Fe(II)-O₂ activation and subsequent C-S bond formation. At 10 °C, *Cth*EgtB exhibited a clear pH dependence, with maximal catalytic activity observed at pH 7.0 (Fig. 4A). Reaction rates decreased upon increasing the pH to 8.0, the condition most commonly employed in prior *Cth*EgtB studies [9, 10], and we observed a progressive reduction in activity from pH 9.0 to pH 10.0 as well. Within the buffer conditions used here, this trend is consistent with catalysis being governed primarily by protonation equilibria within the active site, potentially involving second-sphere residues, substrate functional groups, or proton-transfer networks that modulate Fe(II)/O₂ reactivity as has been previously hypothesized [17, 26]. Notably, the pH optimum for catalytic activity coincides with the previously determined pH of maximal thermal stability for *Cth*EgtB, suggesting that near-neutral conditions simultaneously support structural integrity and efficient catalysis [21]. 

We also studied the impact of temperature on reactivity by monitoring the reaction of Fe(II)-*Cth*EgtB + L-Cys + TMH at pH 7.0, 8.0, 9.0 and 10.0, and across the temperature range from 10 °C to 40 °C (Fig. 4B). *Cth*EgtB displayed the highest activity and greatest thermal responsiveness at pH 7.0 **(**Fig. 4B, black**).** At pH 8.0 and pH 9.0, rates were similar between 10 and 20 °C but diverged at higher temperatures, with pH 8.0 outperforming pH 9.0 at temperatures above 30 °C (Fig. 4B, red and blue). Reactions conducted at the highest pH studied, pH 10.0, displayed uniformly reduced activity and minimal rate enhancement with increasing temperature, with little to no gain in initial rate between 30 and 40 °C (Fig. 4B, green). We hypothesize that the behavior at pH 10.0 is due to destabilization of the catalytic ensemble via subtle perturbations to Fe(II) coordination, protonation state mismatches, or local unfolding, that deviate from the expected Arrhenius behavior. Because effective pKₐ values of ionizable residues and metal-bound intermediates shift with temperature, the protonation state that optimally supports O₂ activation and C-S bond formation is also expected to change. Importantly, previously reported far-UV CD measurements reveal no pH-dependent differences in secondary structure at comparable temperatures, indicating that the observed kinetic trends arise from local active-site equilibria rather than global unfolding [21]. 

Because different buffers were required to cover the full pH range, these data are interpreted as pH-dependent kinetic trends measured under the stated buffer conditions rather than as a complete dissection of buffer-specific effects. All buffers were used at the same concentration, 20 mM, and were prepared using the same anaerobic workflow. These stopped-flow experiments build on our prior biophysical characterization of *Cth*EgtB, in which pH-specific buffers and universal buffer conditions produced the same overall structural trends across pH 4.7–10.0, supporting the interpretation that the present kinetic trends are not primarily driven by buffer identity.

Collectively, these results define a narrow but robust operating window for *Cth*EgtB that is jointly governed by pH and temperature. Conditions near pH 7.0 maximize catalytic efficiency and thermal resilience, while moderately alkaline conditions (pH 8.0) support enhanced temperature responsiveness. The pH- and temperature-dependent rate profiles provide a qualitative framework for estimating apparent pKₐ values associated with catalytically relevant residues or metal-oxygen intermediates, and future analyses could further resolve how protonation equilibria reshape the reaction barrier for *Cth*EgtB-mediated sulfoxide formation.

### Retention of the N-Terminal His-Tag Reduces Activity and Stability of *Cth*EgtB

Affinity tags such as 6xHis have been shown to influence metal binding, protein stability, oligomerization, and kinetics. For this reason, tag removal is often preferred when studying non-heme iron enzymes. However, *Cth*EgtB has been characterized across studies using different construct designs and assay conditions, including differences in affinity-tag retention (6xHis), pH, reducing agents, oxygen availability, and pET vector. Therefore, we sought to determine whether retention of the N-terminal 6xHis tag measurably alters *Cth*EgtB behavior under the same anaerobic stopped-flow conditions used in this work. The purpose of this comparison was not to establish a general rule for metalloenzymes, but to determine whether construct identity measurably affects *Cth*EgtB activity under the same controlled anaerobic conditions used throughout this study. 

**Fig. 5:**
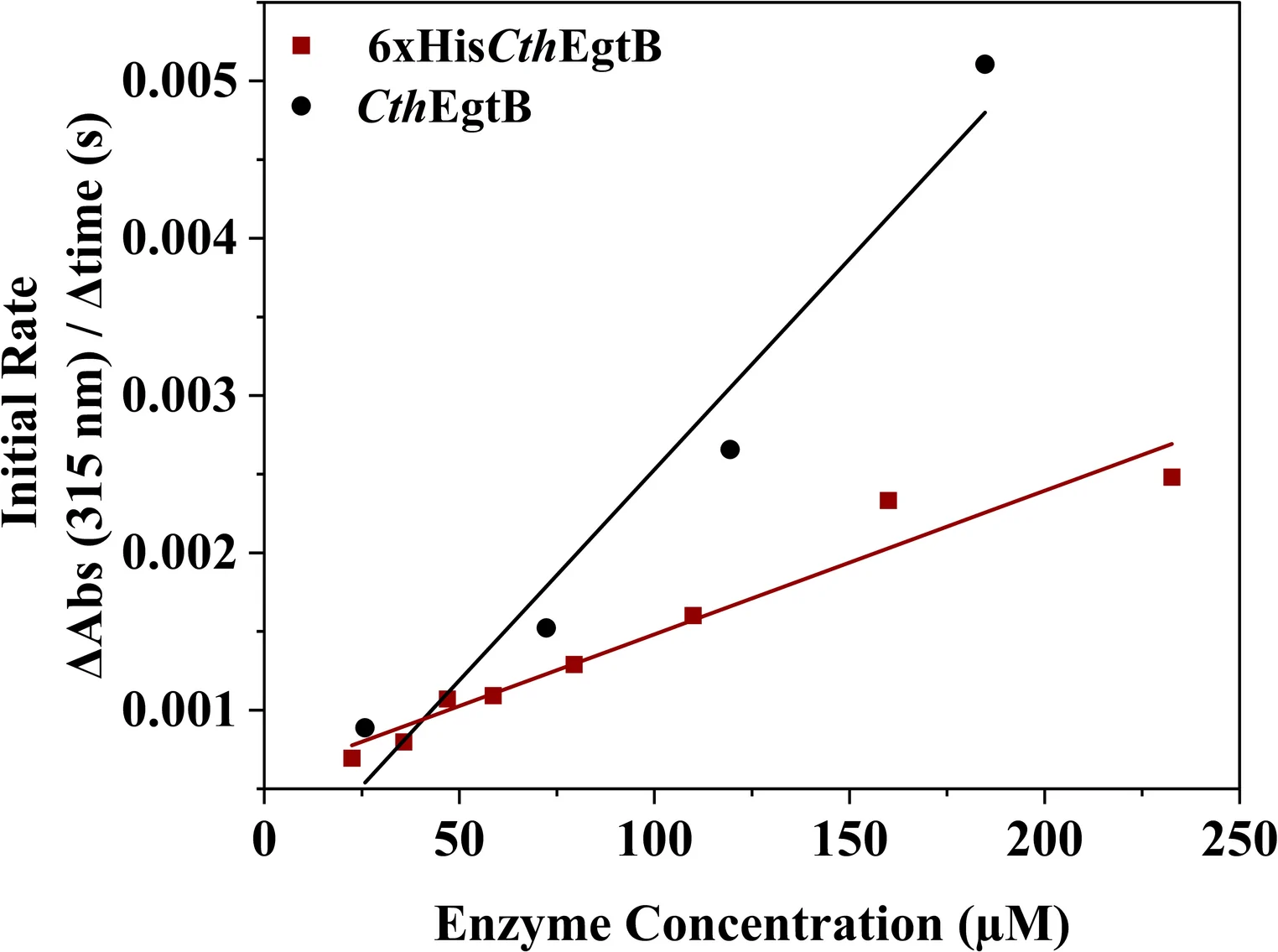
Comparison of catalytic activity of native *Cth*EgtB (black circles) and 6xHis*Cth*EgtB (red squares). Initial reaction velocities were measured by stopped-flow absorbance spectroscopy at the product’s λ_max_ under identical conditions (20 mM HEPES, pH 8.0, 10 °C). Reaction velocity (ΔA/Δt) is plotted as a function of enzyme concentration for both constructs

To evaluate the effect of the N-terminal tag on catalysis, cleaved *Cth*EgtB and 6xHis*Cth*EgtB were compared directly using stopped-flow UV–visible spectroscopy under identical conditions (20 mM HEPES, pH 8.0, 10 °C). Reaction-associated absorbance changes were monitored at 315 nm, and initial velocities were determined across a range of enzyme concentrations (Fig. 5). Across all concentrations tested, 6xHis*Cth*EgtB displayed consistently lower activity than cleaved *Cth*EgtB, reaching only ~ 61% of the native enzyme reaction velocity at equivalent enzyme concentration. At higher concentrations, the His-tagged construct also showed visible aggregation, which prevented reliable collection of additional high-concentration kinetic data under these reaction conditions.

These results demonstrate that retention of the N-terminal 6xHis tag measurably compromises *Cth*EgtB performance under the conditions examined. Importantly, these data do not establish the molecular origin of the reduced activity. Direct metal-occupancy measurements were not performed in the present study, and we therefore do not assign the reduced activity specifically to competition between the His tag and the Fe(II) active site. However, the lower activity of 6xHis*Cth*EgtB may reflect one or more construct-dependent effects, including altered oligomeric behavior, increased aggregation propensity, changes in substrate access, or subtle perturbations to active-site dynamics.

This interpretation is consistent with our previous biophysical characterization of *Cth*EgtB, where retention of the N-terminal 6xHis tag decreased thermal stability, altered oligomeric behavior, and reduced chemical robustness relative to the cleaved enzyme [21]. Taken together, the kinetic and biophysical data indicate that construct identity measurably affects *Cth*EgtB behavior. The value of this comparison is not to make a broad claim about 6xHis tags in all metalloenzymes, but instead to comment specifically about the relevance of affinity tags in studies of *Cth*EgtB. However, kinetic and mechanistic comparisons across literature should consider whether the enzyme was studied as a cleaved or His-tagged construct, particularly when the enzyme of interest utilizes a metal and is oligomeric.

## Conclusion

This work defines how metal loading, substrate binding, oxygen availability, and solution conditions (pH and temperature) collectively govern the stability and reactivity of the non-heme Fe(II) sulfoxide synthase *Cth*EgtB. Far-UV CD and thermal denaturation experiments establish that *Cth*EgtB maintains a predominantly α-helical, tetrameric fold across all catalytic states, and that Fe(II) and ordered substrate binding progressively stabilize the enzyme. Complementary nanoDSF, SLS, and DLS measurements reveal a multiphase unfolding pathway in which subtle pre-cooperative structural rearrangements and higher-order oligomer formation precede global unfolding and aggregation. The present work also highlights the utility of visible CD spectroscopy to provide a direct window into changes in the iron ligand field of *Cth*EgtB upon substrate binding and stepwise exposure of the Fe(II)-*Cth*EgtB + L-Cys + TMH complex to O₂. Future experimental optimization to slow the reaction of *Cth*EgtB and increase the yield of reaction intermediates, combined with visible CD spectroscopy may provide a viable method to track the formation of oxygenated Fe species and product. 

Stopped-flow absorption kinetics of rigorously anaerobic Fe(II)-*Cth*EgtB + L-Cys + TMH mixed with oxygenated buffer show that the initial rate of the reaction-associated 315 nm signal increases with dissolved O₂ concentration [40]. Orthogonal LC-MS/HPLC analysis confirms that these O₂-initiated reaction conditions produce hercynylcysteine sulfoxide, while anaerobic controls do not. Product formation begins within the instrument dead time and proceeds with a smooth, monotonic increase in the absorbance at 315 nm, with no detectable Fe-O₂ or Fe-OOH intermediates under the conditions studied. When combined with the visible CD data, our kinetic data support a unified mechanism in which ES is structurally and electronically poised for reaction involving coupled O₂ activation and C-S bond formation. The kinetic dependence on pH and temperature further refines this mechanistic picture, leading us to identify neutral pH (~ 7.0) as being optimal to maximize catalytic rate, thermal responsiveness, and thermal stability. Finally, direct comparison of native *Cth*EgtB with 6xHis*Cth*EgtB demonstrates that retention of the affinity tag significantly compromises both activity and stability. These findings, together with previously observed differences in thermal and oligomeric properties, challenge the assumption that His-tagged constructs faithfully reproduce the behavior of the native enzyme and call for caution in utilizing prior mechanistic work based solely on tagged variants to calibrate in silico models. Overall, this study shows that *Cth*EgtB operates as a structurally robust, finely tuned catalyst in which metal loading, ordered substrate binding, oxygen availability, pH/temperature, and construct design work together to define catalytic throughput and mechanistic behavior. Future experiments leveraging slow substrates in combination with the methods described here may offer additional handles to probe, trap, and characterize key intermediates in the catalytic cycle.

## Supplementary Information

Below is the link to the electronic supplementary material.

Supplementary Material 1

## Data Availability

Data related to this work (spectra) are provided within the manuscript or the associated supporting information file. Raw data sets (spectra) are available upon request.

## Declarations

### Accession Codes

This manuscript describes expression, purification, biochemical assays and spectroscopic studies of UniProt entry G2LET6_CHLTF.
