# Supplementary material for: Insights into the pH-dependent reactivity and substrate induced active-site reorganization of the non- heme iron enzyme CthEgtB
Source: J Biol Inorg Chem. 2026 Jul 30;31(6):507–19. doi: 10.1007/s00775-026-02167-8 (PMC13645936; doi:10.1007/s00775-026-02167-8)
Supplement: Supplementary file 1 [file 775_2026_2167_MOESM1_ESM.pdf]

Supplementary Information

Insights into the pH-dependent Reactivity and Substrate Induced Active-Site Reorganization of the Non-Heme Iron Enzyme *Cth*EgtB

Kassidy W. Rodriguez[1], Phuong Hong Ngoc Tao[2,3], Kiran Kurmi[2,3], *Katlyn K. Meier*[1]*

Affiliations

[1] University of Miami, Department of Chemistry, Coral Gables, FL, 33146, United States

[2]Department of Molecular and Cellular Pharmacology; Sylvester Comprehensive Cancer Center, University of Miami Miller School of Medicine, Miami, FL 33136, USA

[3]The Frost Institute for Chemistry and Molecular Science, University of Miami, Miami, FL 33146, USA

Corresponding Author Email: kmeier@miami.edu

Table of Contents

	Content	Page
	Detailed Materials and Methods	**S4-S8**
**Figure S1**	Far-UV circular dichroism (CD) spectra of *Cth*EgtB collected at 20 °C (200–250 nm).	**S9**
**Table S1**	Melting temperatures (T_m) determined by far-UV circular dichroism (CD) for *Cth*EgtB following sequential Fe(II), substrate (L-Cys, TMH), and O_2 exposure.	**S10**
	Multiphase Thermal Unfolding of CthEgtB Reveals Distinct Structural Transitions Probed by CD and nanoDSF.	**S11**
**Figure S2**	Differential thermal unfolding behavior of *Cth*EgtB monitored by distinct far-UV CD transitions.	**S12**
**Table S2**	NanoDSF-derived melting temperatures (T_m) for *Cth*EgtB measured using a Prometheus Panta instrument under the indicated conditions.	**S12**
**Figure S3**	Multimodal thermal denaturation of *Cth*EgtB monitored by nanoDSF, SLS, and DLS.	**S13**
**Figure S4**	Visible absorption and CD spectra of *Cth*EgtB are sensitive measures of ligand-field changes during substrate binding and the reaction with O_2.	**S14**
**Figure S5**	Stopped-flow UV-vis controls and data processing for O_2-dependent *Cth*EgtB catalysis.	**S15**
**Figure S6**	LC-MS analysis confirms TMH consumption and O_2-dependent hercynylcysteine sulfoxide formation..	**S16**

	References	**S17**

Detailed Materials and Methods

CthEgtB construct, expression, and purification. Details regarding the enzyme construct design as well as expression and purification procedures have been reported previously, although with slight modifications.[1] Briefly, the gene encoding EgtB from *Chloracidobacterium thermophilum* (*Cth*EgtB, UniProt ID: G2LET6_CHLTF) was cloned into a pET28a vector by GenScript and verified by sequencing (Azenta Life Sciences). The construct encoded an N-terminal 6xHis tag followed by a Tobacco Etch Virus (TEV) protease cleavage site. For protein expression, BL21(DE3) *E. coli* cells harboring the plasmid were grown in 1 L cultures at 37 °C and 220 rpm until an OD_{600} of 0.5 was reached, at which point protein expression was induced with 1 mM isopropyl β-D-1-thiogalactopyranoside (IPTG). The N-terminally tagged enzyme (6xHis*Cth*EgtB) was purified using Ni^{2+}-NTA affinity chromatography. To remove the affinity tag, TEV protease (prepared in house from plasmid, pDZ2087, a gift from David Waugh; Addgene plasmid #92414; http://n2t.net/addgene:92414; RRID: Addgene_92414)[2] was added at a 1:100 molar ratio (TEV:6xHis*Cth*EgtB), and the reaction mixture was subjected to a second Ni^{2+}-NTA purification step to purify cleaved *Cth*EgtB from the tag and protease.

Preparation of Anaerobic Enzyme Samples. Following purification of either 6xHis*Cth*EgtB or *Cth*EgtB, the enzyme was dialyzed overnight (4 °C) against EDTA Buffer (50 mM sodium phosphate, 50 mM NaCl, 25 mM EDTA, pH 8.0) to remove metals present from expression and scale-up. The enzyme was then dialyzed into Buffer 1 (20 mM HEPES, pH 8.0) to eliminate residual phosphate and EDTA. An additional overnight dialysis was performed in Buffer 1 (Chelex® treated buffer). This preparation was used for subsequent addition of Fe(II) (ammonium iron(II) sulfate hydrate, Puratonic™, 99.999%) to enzyme samples. On the day of each experiment, the enzyme in Chelex® treated Buffer 1 was sterile filtered (0.22 µm filter) and adjusted to the desired concentration prior to spectroscopic measurements. The metal-free enzyme solution was transferred into a 25 mL scintillation vial fitted with a septum sealed cap. Ultra-high purity (UHP) argon gas was flowed through the headspace of the vial while the solution was gently stirred on ice (60 rpm) at 3 psi for 1 h to displace residual oxygen. The sealed vial was then transferred into an anaerobic glove box maintained at <0.1 ppm O_2 for subsequent use. A 10 µL aliquot of the enzyme was removed from the glovebox to determine concentration by absorbance at 280 nm (NanoDrop One microvolume UV–Vis spectrophotometer; Thermo Scientific) prior to substrate addition (TMH, L-Cys, or O_2) and spectroscopic

measurement. All samples in this study correspond to anaerobically prepared *Cth*EgtB or its 6xHis-tagged variant (6xHis*Cth*EgtB).

*Circular Dichroism (CD). Cth*EgtB was dialyzed into 20 mM phosphate, 20 mM NaCl, pH 8.0 or Buffer 1 for far-UV or visible CD, respectively. Far-UV and visible CD spectra were recorded on an Applied Photophysics Chirascan V100 spectrometer equipped with a Xe lamp and large-area avalanche photodiode detector. Far-UV and visible CD data were collected using 2 or 10 mm pathlength 6Q quartz cuvettes (Starna Cells), respectively, with septum sealed caps. Thermal unfolding experiments were performed in triplicate in the far-UV region (200–250 nm) over a temperature range of 20–90 °C, with the temperature increased in 2 °C increments using a Peltier temperature controller coupled to a circulating water bath. Enzyme samples (2-3 μM) were equilibrated for 2 min at each temperature prior to measurement. Thermal unfolding data were analyzed using the Global3 software package (Applied Photophysics). A global fit of the data collected between 200 and 250 nm was performed, assuming a single transition and single baseline correction. The fitted data were used to generate sigmoidal unfolding curves describing the transition from folded (F) to unfolded (U) states and to extract the transition midpoint (T_m, where 50% of the protein is unfolded).

Visible CD data were collected at 20 °C over the wavelength range from 300 nm to 1100 nm in step sizes of 1 nm with a 0.5 nm bandwidth. Peak maxima were determined in MATLAB using a quadratic peak fitting procedure and all analyses were performed using scripts based solely on core MATLAB functions. For each spectrum, a wavelength window encompassing the transition was isolated using logical indexing. A preliminary peak estimate was obtained by locating the maximum ellipticity within this window. To refine the peak position beyond the experimental step size, a second-order polynomial (quadratic) was fitted locally to the CD signal at the rough peak maximum. Quadratic coefficients were obtained via least-squares regression using MATLAB's polyfit function:

$$y(\lambda) = a\lambda^2 + b\lambda + c \qquad \textbf{(1)}$$

The analytically determined vertex of the parabola provided the refined peak wavelength according to **(2)**.

$$\lambda_{max} = -\frac{b}{2a} \qquad \textbf{(2)}$$

This method was used to obtain the final reported λ_{max} values for all spectra.

Prometheus Panta (NanoTemper). Enzyme samples were sterile filtered and centrifuged at 15,000 × g for 10 min in the glovebox. Enzyme samples loaded into Prometheus-compatible capillaries ranged from 2-4 μM. Prometheus-compatible capillaries were aligned in the capillary tray, secured using the magnetic lid, and sealed by applying two successive layers of NanoTemper sealing paste using the manufacturer's applicators, with a brief drying period between applications. This treatment enabled us to maintain our anaerobic samples. Sealed capillaries were fully dried before being removed from the glovebox for measurement in the Prometheus Panta instrument.

Stopped-Flow Kinetic Measurements. Stopped-flow experiments were performed using an SX20 stopped-flow spectrometer (Applied Photophysics) equipped with absorbance detection. Absorbance measurements were collected using a 10 mm optical pathlength with an instrument dead time of 2 ms. An anaerobic accessory (Applied Photophysics) was used to facilitate low-oxygen handling during kinetic measurements. Time-resolved UV–visible spectra were acquired using a directly coupled photodiode array (PDA) detector (Applied Photophysics), enabling simultaneous collection of full absorbance spectra (270-725 nm) following a single stopped-flow drive. Excitation was provided by a xenon arc lamp coupled to a programmable monochromator, and the instrument temperature was maintained using a programmable recirculating water bath.

Reactions were initiated by rapid mixing of oxygen-saturated buffer (*see next subsection*) with anaerobically prepared enzyme–substrate complexes. Specifically, syringe 1 contained Fe(II)–*Cth*EgtB+TMH+L-Cys prepared under strictly anaerobic conditions in Buffer 1, while syringe 2 contained oxygenated Buffer 1 at specific dissolved O_2 concentrations. All solutions were equilibrated to the experimental temperature prior to mixing. Data analysis and visualization were performed using the Pro-Data software (Applied Photophysics). Global kinetic analysis of time-resolved spectral data and extracted single-wavelength absorbance traces was carried out using Pro-K IV software (Applied Photophysics). At least three independent shots were averaged for each condition to improve signal-to-noise, and experiments were conducted for a minimum of three enzyme concentrations.

Buffer Selection for pH-dependent Measurements. For pH-dependent stopped-flow measurements, all reactions were performed in 20 mM buffer to maintain buffering capacity while minimizing potential buffer–enzyme interactions. MOPS was used for pH 6.5–7.0, HEPES for pH 7.5–8.0, and glycine for pH 9.0–10.0. Buffers were selected based on their effective buffering ranges and compatibility with anaerobic Fe(II)-*Cth*EgtB samples. Phosphate buffer was not used because phosphate-containing Fe(II)-*Cth*EgtB samples were prone to precipitation under the high enzyme

concentrations (0.5-2 mM) and anaerobic Fe(II)-reconstitution conditions required for visible CD measurements. All buffers were prepared at the same concentration and treated using the same anaerobic preparation workflow prior to use. Because buffer identity can influence enzymatic rates, pH-dependent stopped-flow data are interpreted as kinetic trends measured under the stated buffer conditions. Further, these experiments were designed to build on our prior biophysical characterization of *Cth*EgtB, in which pH-specific buffers and universal buffer conditions produced the same overall structural trends across pH 4.7–10.0. Thus, the structural baseline used to interpret the present kinetic measurements is not expected to arise primarily from buffer identity.

Oxygen Saturated Buffer Preparation and Concentration Determination. To quantify the O_2 concentration in oxygenated buffer solutions, we employed previously established methods[3] using a methyl viologen–based colorimetric assay in which reduced methyl viologen (MV^{+}•) is oxidized stoichiometrically by O_2, resulting in a measurable loss of absorbance at 605 nm. All solutions were prepared using anaerobic water. Stock solutions of 10 mM methyl viologen, 10 mM potassium ferricyanide, and 10 mM sodium dithionite were prepared in anaerobic water; sodium dithionite solutions were freshly prepared due to rapid decomposition. The dithionite stock concentration was standardized by monitoring the reduction of potassium ferricyanide. A 1 mM ferricyanide solution was prepared by mixing 100 μL of 10 mM ferricyanide with 900 μL anaerobic water, loaded into syringe 1 of the stopped-flow spectrophotometer, with anaerobic water loaded into syringe 2. After flushing and blanking the instrument with anaerobic water, the absorbance spectrum of the mixed ferricyanide solution (0.5 mM post-mix) was collected. A 0.1 mM sodium dithionite solution was then prepared by diluting 10 μL of the 10 mM dithionite stock into 990 μL anaerobic water and loaded into syringe 2 to measure the reduction of ferricyanide over time (complete reduction required at least 3000 s). The concentration of dithionite was calculated from the change in ferricyanide absorbance using the Beer–Lambert law with $\varepsilon_{405} = 1.04\ mM^{-1}\ cm^{-1}$, which reflects the wavelength used in the established oxygen-quantification protocol. Reduced methyl viologen was then generated by mixing equimolar methyl viologen and sodium dithionite to yield a 2.5 mM MV^{+}• stock (0.125 mL of 0.1 M methyl viologen + 0.125 mL of 0.1 M dithionite + 4.75 mL anaerobic water). To generate an anaerobic control sample, 50 μL of the reduced MV^{+}• stock was mixed with 1 mL anaerobic water containing 2 mM dithionite, and the absorbance spectrum was recorded anaerobically using the stopped-flow instrument (syringe 1: reduced MV^{+}• + dithionite; syringe 2: anaerobic water). Separately, an experimental sample was prepared by mixing 50 μL reduced MV^{+}• with 1 mL anaerobic water or buffer without added dithionite. In this case, any residual dissolved O_2 oxidized MV^{+}• during the measurement. The change in absorbance

between the anaerobic control and the sample at 605 nm (ε_{605} = 13.7 mM^{-1} cm^{-1}) was used to quantify the amount of $MV^{+\bullet}$ oxidized. Because methyl viologen is oxidized in a 1:1 stoichiometry with molecular oxygen, the concentration of oxidized $MV^{+\bullet}$ corresponds directly to the concentration of dissolved oxygen in the buffer. When oxygen levels were sufficiently high to fully oxidize the reduced methyl viologen, MV concentrations were increased to maintain quantifiable absorbance changes. This method allows sensitive determination of oxygen contamination and validation of O_2-free buffers prepared for anaerobic enzymology or stopped-flow experiments.

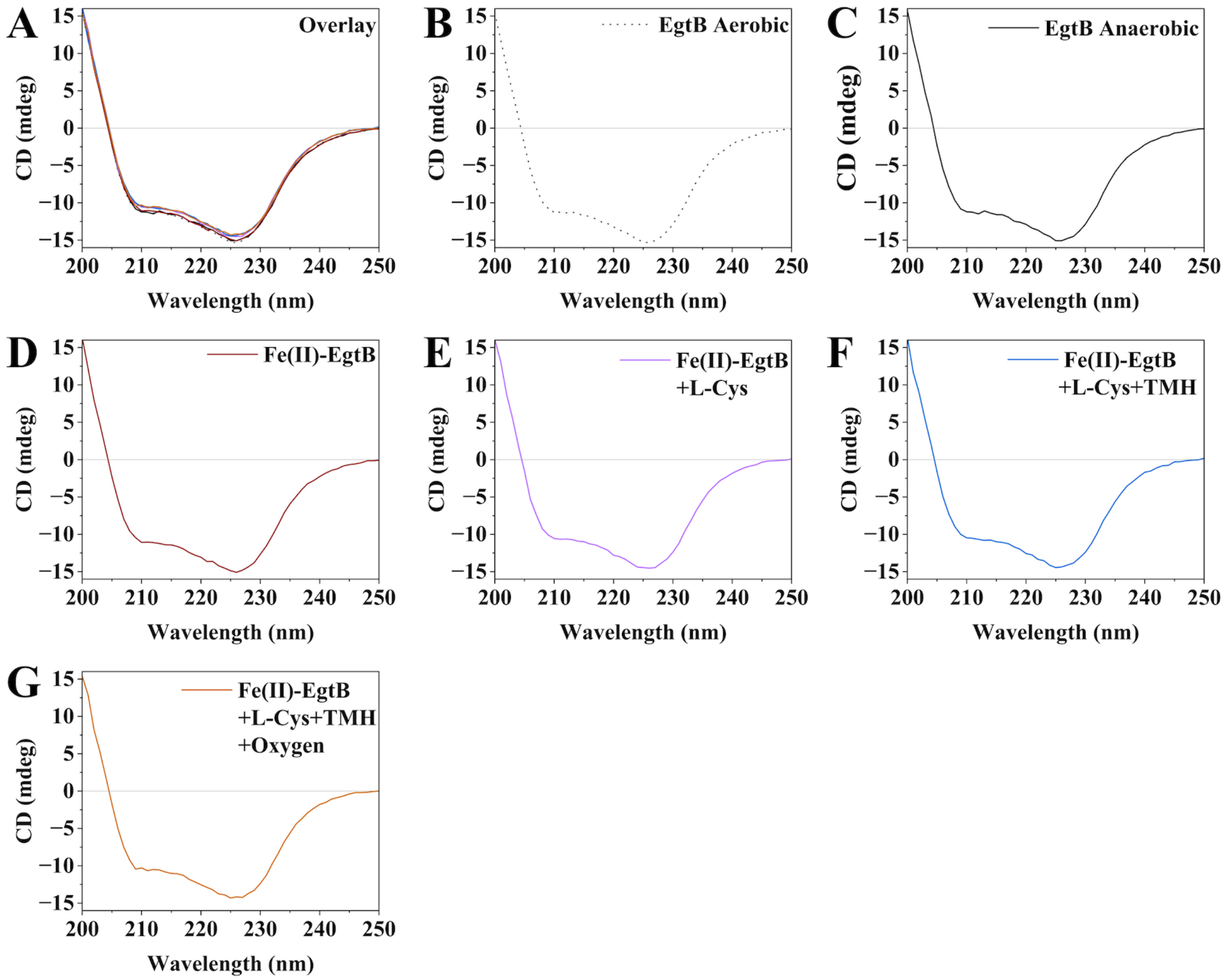

Fig. S1 Far-UV circular dichroism (CD) spectra of *Cth*EgtB collected at 20 °C (200–250 nm). Spectra were recorded in 20 mM dibasic phosphate, 20 mM NaCl, pH 8.0, with 1.5–2 µM *Cth*EgtB. (A) All conditions exhibited pronounced minima at 211 and 227 nm, consistent with a predominantly α-helical secondary structure. Identical spectra were observed for the native enzyme (B) before and (C) after anaerobic preparation, following sequential addition of (D) Fe(II), (E) L-cysteine, and (F) trimethylhistidine (TMH), and (G) after exposure to O_2 in the presence of the sulfoxide product, indicating that substrate binding and turnover do not induce global changes in *Cth*EgtB secondary structure

Table S1 Melting temperatures (T_m) determined by far-UV circular dichroism (CD) for *Cth*EgtB following sequential Fe(II), substrate (L-Cys, TMH), and O_2 exposure

**Sample**	**T_m (°C)**	**Standard Deviation (n = 3)**
***Cth*EgtB**	72.36	0.14
**Fe(II)-*Cth*EgtB**	73.62	0.11
**Fe(II)-*Cth*EgtB + L-Cys + TMH**	75.62	0.10
**Fe(II)-*Cth*EgtB + L-Cys + TMH + O_2**	74.36	0.17
***Cth*EgtB + L-Cys + TMH**	72.36	0.11

Multiphase Thermal Unfolding of CthEgtB Reveals Distinct Structural Transitions Probed by CD and nanoDSF. Across all CD thermal unfolding experiments, *Cth*EgtB did not unfold as a single cooperative unit but instead exhibited multiphase behavior (**Figure S2**). Loss of ellipticity at ~227 nm, corresponding to the n→π* transition associated with α-helical elements implicated in tetramer stabilization, occurred earlier and more gradually than changes at ~209 nm, which report on more tightly packed, monomer-localized α-helical secondary structure. These observations indicate that quaternary-associated or flexible helical regions are perturbed prior to cooperative unfolding of the protein core.

Thermal denaturation was further studied using a Prometheus Panta instrument (NanoTemper), which simultaneously monitors intrinsic fluorescence (nanoDSF), static light scattering (SLS), and dynamic light scattering (DLS). Across all conditions tested, including anaerobically prepared enzyme, Fe(II)-bound enzyme with and without substrates, and samples exposed to O_2, the nanoDSF-derived melting temperatures were statistically indistinguishable, yielding an average T_m of 71.89 ± 0.18 °C (**Table S2**). Thus, metal binding, substrate binding, and anaerobic handling do not appreciably alter the tertiary structure surrounding aromatic residues. Despite the absence of T_m shifts by nanoDSF, the fluorescence traces exhibited a gradual, linear increase between ~20 and 60 °C prior to cooperative unfolding (**Figure S3**). This pre-transition parallels the early CD-detected loss of ellipticity at 227 nm, suggesting that both techniques capture subtle, non-cooperative structural rearrangements, potentially involving tetramer packing or flexible helical regions. At approximately 68 °C, all samples exhibited a sharp decrease in the F_{350}/F_{330} fluorescence ratio, marking the onset of cooperative global unfolding. This event was immediately followed by increased turbidity detected by SLS (**Figure S3C),** indicating that aggregation occurs downstream of unfolding. Consistent with this interpretation, DLS measurements revealed the formation of larger hydrodynamic species with increasing temperature (**Figure S3D**), consistent with transient higher-order assemblies prior to irreversible aggregation.

Overall, the combined CD, nanoDSF, SLS, and DLS data define a multiphase thermal denaturation pathway for *Cth*EgtB comprising early structural rearrangements, cooperative unfolding near ~72 °C, and subsequent aggregation. The apparent discrepancy between CD and nanoDSF-derived stabilization reflects the distinct structural features probed by each technique: CD is sensitive to secondary-structure and quaternary packing, whereas nanoDSF primarily reports on local tertiary environments. Consequently, stabilization of α-helical and tetrameric elements by Fe(II) and substrate binding yields measurable CD T_m shifts without altering the nanoDSF T_m.

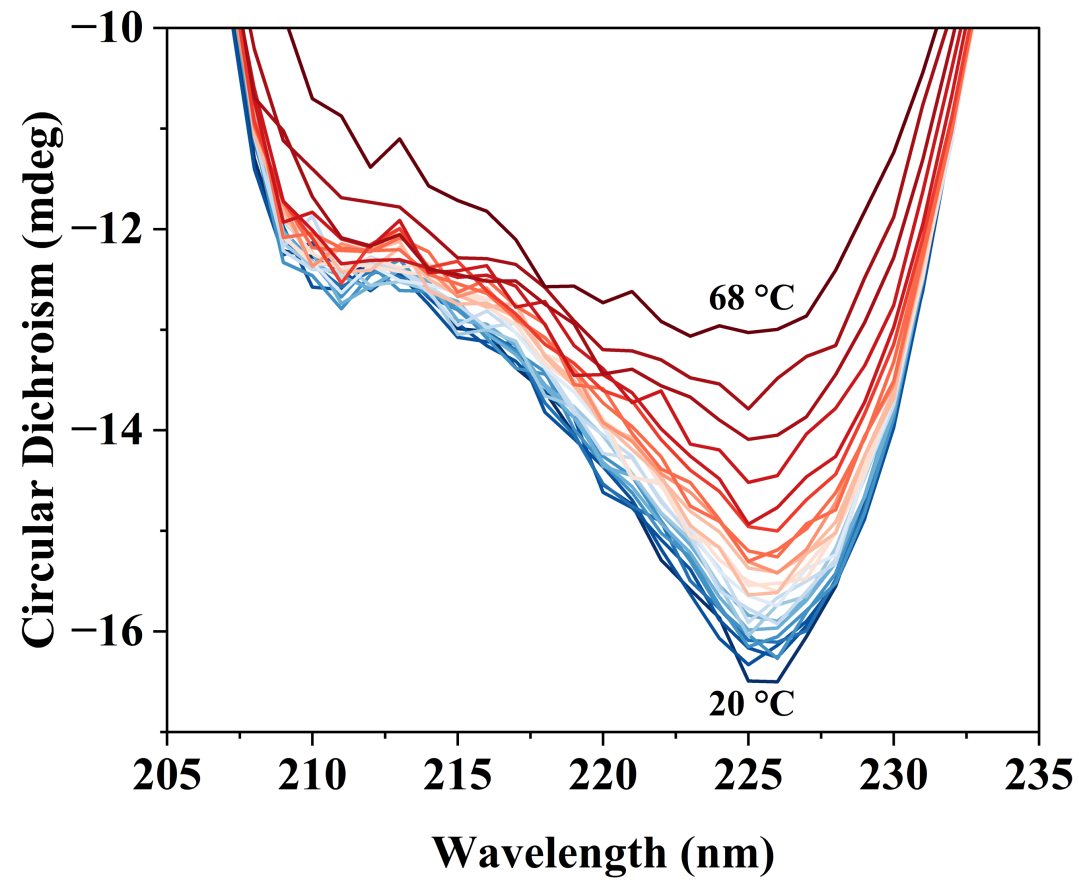

Fig. S2 Differential thermal unfolding behavior of *Cth*EgtB monitored by distinct far-UV CD transitions. Comparison of thermal unfolding profiles monitored at the n→π* and π→π* transitions reveals non-cooperative unfolding behavior in *Cth*EgtB. Structural elements reported by the n→π* transition, associated with α-helical features contributing to tetramer stabilization, destabilize earlier and less cooperatively than those reported by the π→π* transition, which reflects more tightly packed α-helical secondary structure within individual monomers

Table S2 NanoDSF-derived melting temperatures (T_m) for *Cth*EgtB measured using a Prometheus Panta instrument under the indicated conditions

Sample	T_m (°C)	Standard Deviation (n = 3)
***Cth*EgtB**	71.50	0.06
**Fe(II)-*Cth*EgtB**	71.58	0.17
**Fe(II)-*Cth*EgtB + L-Cys + TMH**	71.72	0.13
**Fe(II)-*Cth*EgtB + L-Cys + TMH + O_2**	71.78	0.06
***Cth*EgtB + L-Cys + TMH**	71.59	0.06

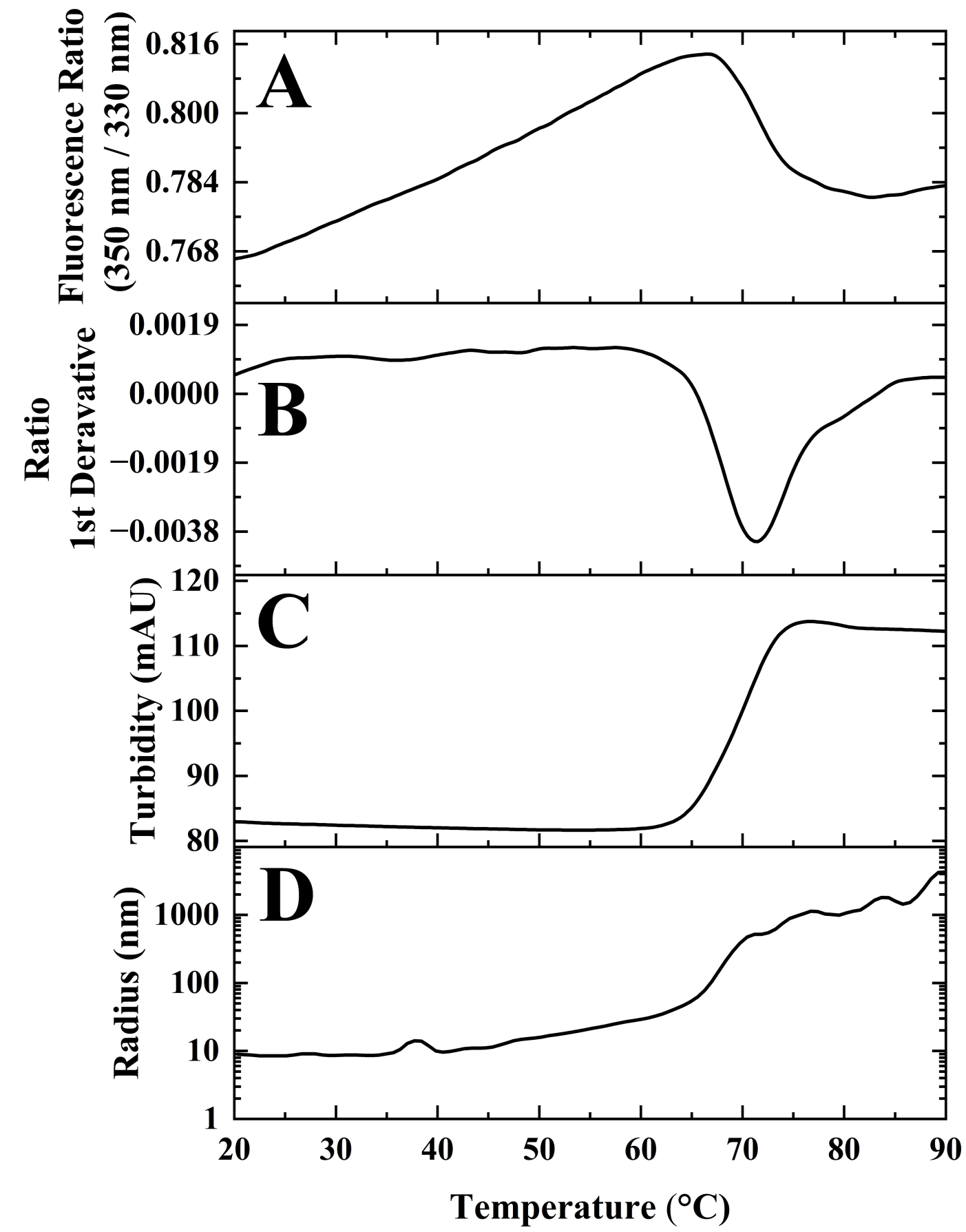

Fig. S3 Multimodal thermal denaturation of *Cth*EgtB monitored by nanoDSF, SLS, and DLS. (A) Temperature-dependent changes in the intrinsic fluorescence ratio (F_{350}/F_{330}) measured by nanoDSF reveal a gradual, linear increase from ~20–60 °C followed by a sharp decrease associated with cooperative unfolding. (B) First derivative of the F_{350}/F_{330} ratio highlighting the cooperative unfolding transition near ~72 °C. (C) Static light scattering (SLS) traces showing increased turbidity at elevated temperatures, indicative of aggregation occurring downstream of global unfolding. (D) Dynamic light scattering (DLS) measurements showing an increase in cumulant radius with temperature, consistent with the formation of higher-order species prior to irreversible aggregation

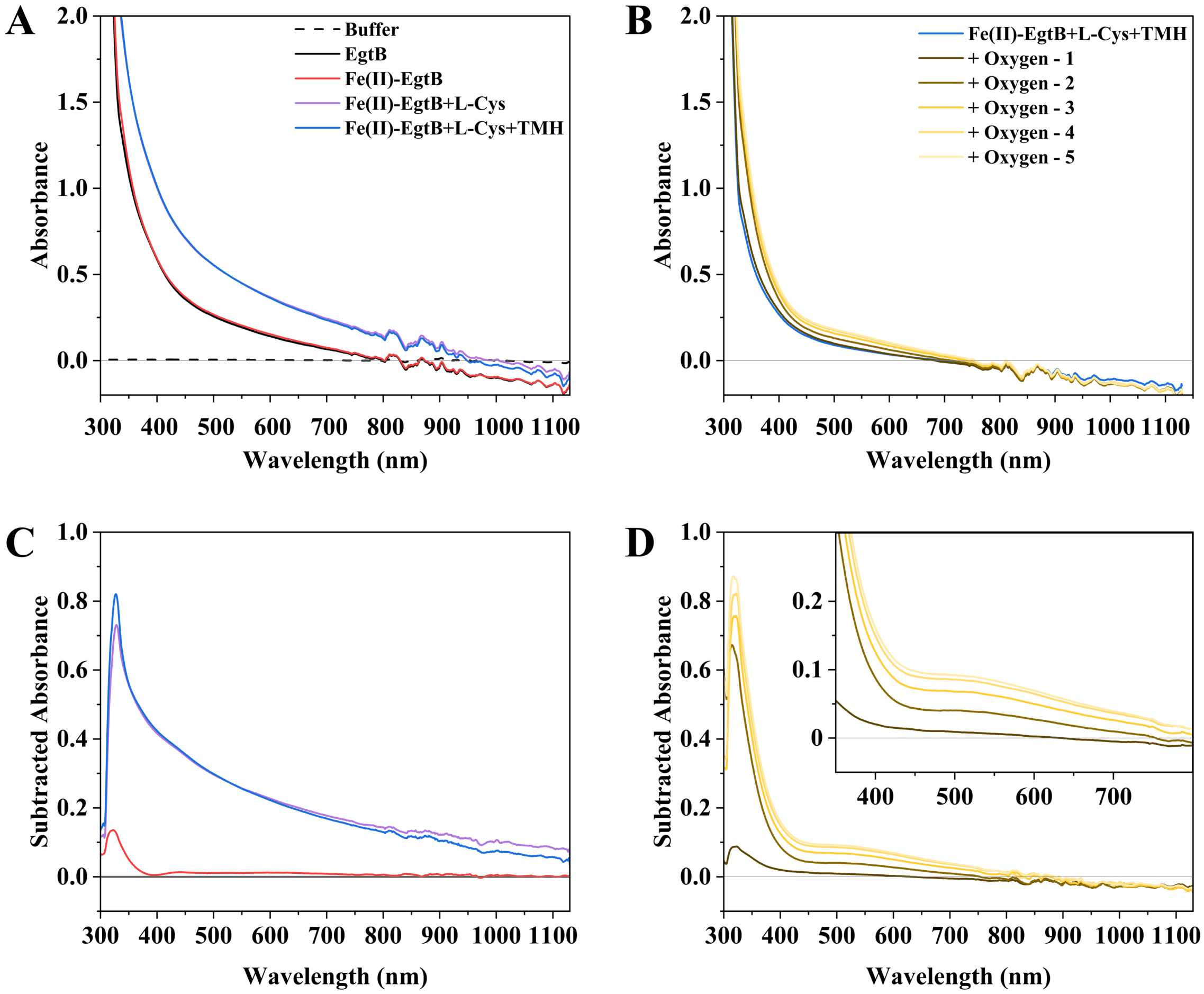

Fig. S4 Visible absorption and CD spectra of *Cth*EgtB are sensitive measures of ligand-field changes during substrate binding and the reaction with O_2. (A) Anaerobic visible absorption spectra of apo-*Cth*EgtB (black; 1.50 mM; 20 °C, 20 mM HEPES, pH 8.0), Fe(II)-*Cth*EgtB (red), and substrate-bound Fe(II)-*Cth*EgtB (purple = L-Cys only; blue = L-Cys + TMH). (B) Visible absorption spectra collected after incremental O_2 exposure (dark to light orange; labeled Oxygen-1 through Oxygen-5) of the preassembled Fe(II)-*Cth*EgtB+L-Cys+TMH complex (blue, 0.70 mM). (C) Subtracted absorption spectra corresponding to panel A. Spectra were collected using 20 mM HEPES, pH 8.0 buffer as a blank, and the apo-*Cth*EgtB spectrum was subtracted to emphasize Fe(II)- and substrate-dependent changes in the visible/near-IR region. (D) Subtracted absorption spectra corresponding to panel B. Spectra were collected using 20 mM HEPES, pH 8.0 buffer as a blank, and the spectrum of the preassembled Fe(II)-*Cth*EgtB+L-Cys+TMH complex prior to O_2 exposure was subtracted to emphasize O_2-dependent spectral changes. Absorption data below ~340 nm are not shown in the main text because the intensity of the raw data exceeded 2 AU in that region. Signal-to-noise also decreases near the long-wavelength detector cutoff (~1150 nm).

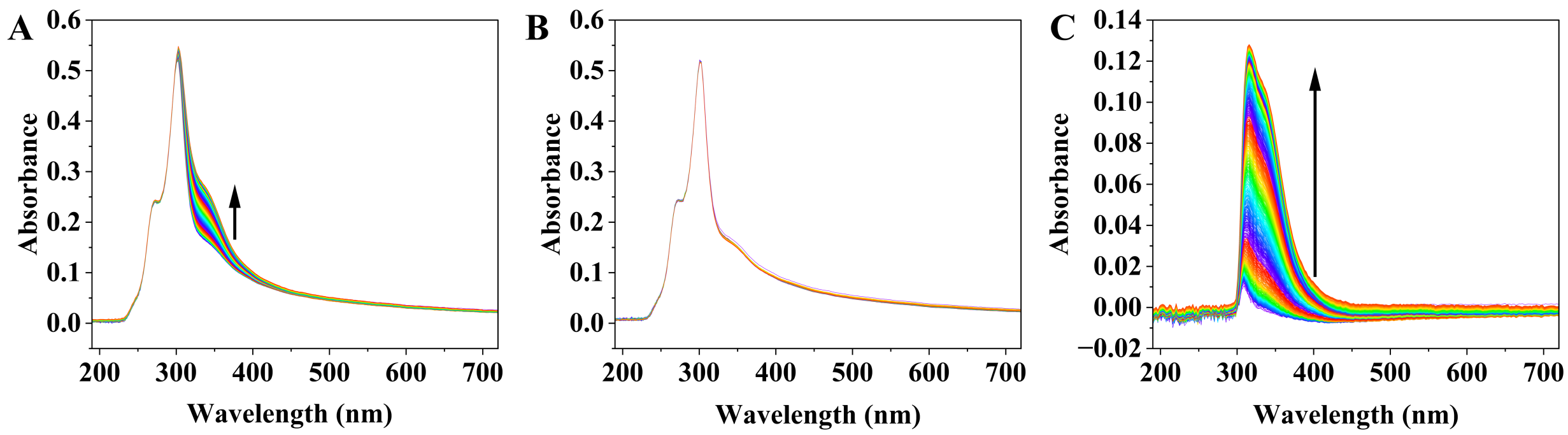

Fig. S5 Stopped-flow UV-vis controls and data processing for O_2-dependent *Cth*EgtB catalysis. (A) Stopped-flow absorbance traces obtained upon mixing anaerobically prepared Fe(II)-*Cth*EgtB+L-Cys+TMH (syringe 1) with O_2-saturated buffer (syringe 2), initiating catalysis. (B) Control experiment in which the same Fe(II)-*Cth*EgtB+L-Cys+TMH complex (syringe 1) was mixed with anaerobic buffer (syringe 2), showing no detectable absorbance change and confirming the strict dependence of product formation on molecular oxygen. (C) Difference spectrum generated by subtraction of the anaerobic control (panel B) from the O_2-initiated reaction (panel A), yielding the absorbance spectrum of the sulfoxide product. All solutions were prepared under strictly anaerobic conditions (<0.1 ppm O_2) prior to mixing, with Fe(II) present at a 1:1 molar ratio relative to enzyme and substrates (L-Cys and TMH) in excess (1:10)

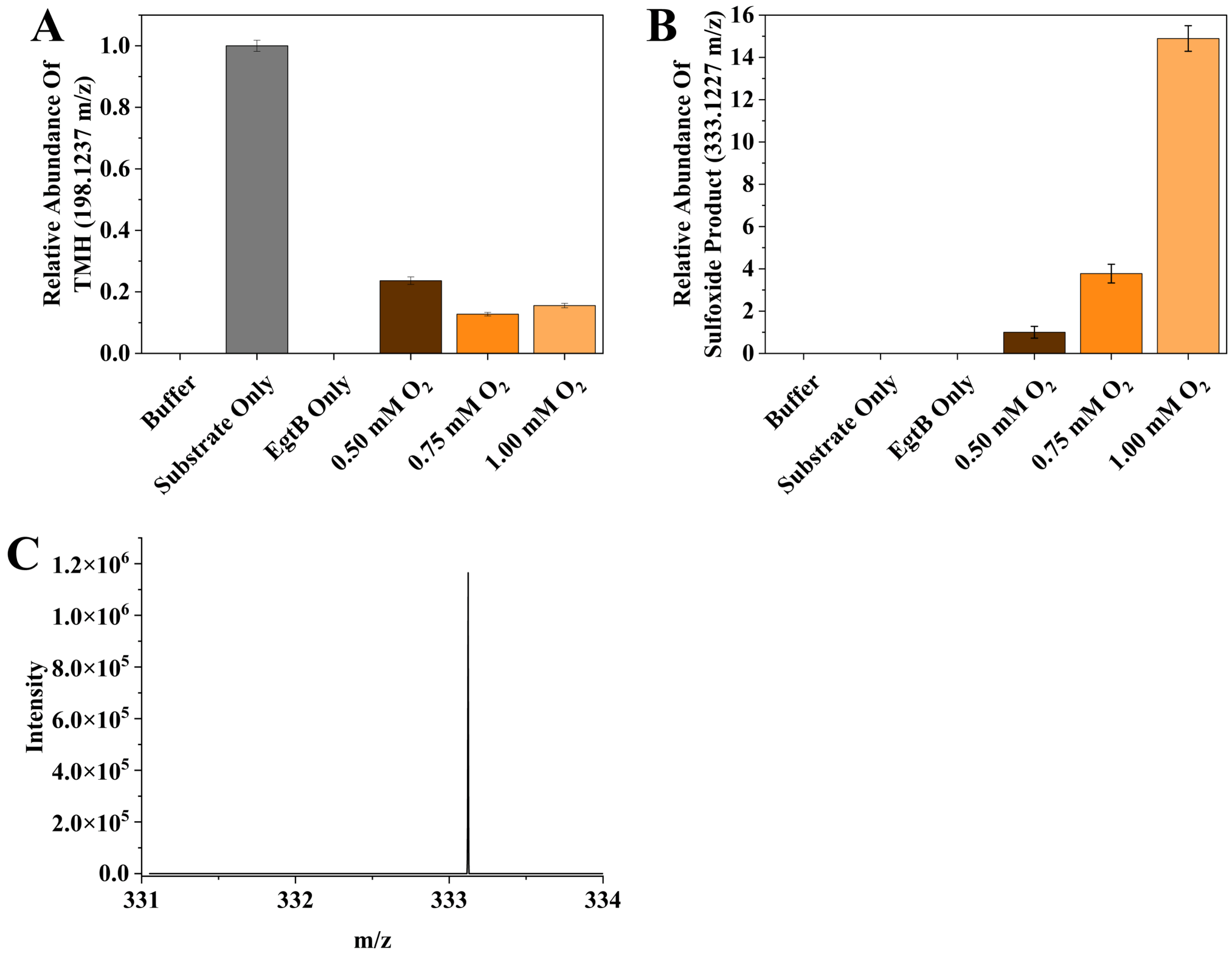

Fig. S6. LC-MS analysis confirms TMH consumption and O_2-dependent hercynylcysteine sulfoxide formation. LC-MS samples were prepared under anaerobic conditions and initiated by mixing Fe(II)-CthEgtB + L-Cys + TMH with O_2-saturated buffer. Reactions were allowed to proceed for 1000 s and were quenched with ice-cold LC-MS-grade methanol. **(A)** Relative abundance of TMH/hercynine detected in positive ion mode using the extracted ion signal for the protonated substrate, $[M+H]^+$, at *m/z* 198.1237. TMH was abundant in the substrate-only control and decreased in the O_2-initiated enzyme reactions, consistent with substrate consumption during turnover. **(B)** Relative abundance of hercynylcysteine sulfoxide product detected using the extracted ion signal for the protonated product, $[M+H]^+$, at *m/z* 333.1227. Product was not detected in buffer-only, substrate-only, or Fe(II)-CthEgtB-only controls, but increased with increasing O_2 availability. The corresponding oxygen concentrations used initiate the reactions (0.50, 0.75, and 1.00 mM O_2) are listed on the x-axes of panels A-B. **(C)** Representative MS1 spectrum showing the product-associated ion at *m/z* 333.1227. Bars in panels A and B represent the mean relative abundance from technical triplicates, and error bars represent standard deviation.

References

1. Rodriguez, K.W. and K.K. Meier, *Defining Factors that Influence the Stability of Tetrameric EgtB from Chloracidobacterium thermophilum: From pH Modulation to Affinity Tag Retention.* Biochemistry, 2025. **64**(16): p. 3526-3534.
2. Raran-Kurussi, S., et al., *Removal of Affinity Tags with TEV Protease.* Methods Mol Biol, 2017. **1586**: p. 221-230.
3. Valentino, H. and P. Sobrado, *Performing anaerobic stopped-flow spectrophotometry inside of an anaerobic chamber.* Methods Enzymol, 2019. **620**: p. 51-88.
